# Enablers and barriers to the implementation of primary health care interventions for Indigenous people with chronic diseases: a systematic review

**DOI:** 10.1186/s13012-015-0261-x

**Published:** 2015-05-22

**Authors:** Odette Gibson, Karolina Lisy, Carol Davy, Edoardo Aromataris, Elaine Kite, Craig Lockwood, Dagmara Riitano, Katharine McBride, Alex Brown

**Affiliations:** 1 Wardliparingga Aboriginal Research Unit, South Australian Health and Medical Research Institute, Adelaide, Australia; 2 University of South Australia, Adelaide, Australia; 3 Joanna Briggs Institute, University of Adelaide, Adelaide, Australia; 4 University of Adelaide, Adelaide, Australia

**Keywords:** Australia, New Zealand, Canada, United States, Primary health care, Intervention, Chronic disease, Indigenous peoples

## Abstract

**Background:**

Access to appropriate, affordable, acceptable and comprehensive primary health care (PHC) is critical for improving the health of Indigenous populations. Whilst appropriate infrastructure, sufficient funding and knowledgeable health care professionals are crucial, these elements alone will not lead to the provision of appropriate care for all Indigenous people. This systematic literature review synthesised international evidence on the factors that enable or inhibit the implementation of interventions aimed at improving chronic disease care for Indigenous people.

**Methods:**

A systematic review using Medical Literature Analysis and Retrieval System Online (MEDLINE) (PubMed platform), Web of Science, Cumulative Index to Nursing and Allied Health Literature (CINAHL), PsycINFO, Excerpta Medica Database (EMBASE), ATSIHealth, Australian Indigenous HealthInfoNet via Informit Online and Primary Health Care Research and Information Service (PHCRIS) databases was undertaken. Studies were included if they described an intervention for one or more of six chronic conditions that was delivered in a primary health care setting in Australia, New Zealand, Canada or the United States. Attitudes, beliefs, expectations, understandings and knowledge of patients, their families, Indigenous communities, providers and policy makers were of interest. Published and unpublished qualitative and quantitative studies from 1998 to 2013 were considered. Qualitative findings were pooled using a meta-aggregative approach, and quantitative data were presented as a narrative summary.

**Results:**

Twenty three studies were included. Meta-aggregation of qualitative data revealed five synthesised findings, related to issues within the design and planning phase of interventions, the chronic disease workforce, partnerships between service providers and patients, clinical care pathways and patient access to services. The available quantitative data supported the qualitative findings. Three key features of enablers and barriers emerged from the findings: (1) they are not fixed concepts but can be positively or negatively influenced, (2) the degree to which the work of an intervention can influence an enabler or barrier varies depending on their source and (3) they are inter-related whereby a change in one may effect a change in another.

**Conclusions:**

Future interventions should consider the findings of this review as it provides an evidence-base that contributes to the successful design, implementation and sustainability of chronic disease interventions in primary health care settings intended for Indigenous people.

**Electronic supplementary material:**

The online version of this article (doi:10.1186/s13012-015-0261-x) contains supplementary material, which is available to authorized users.

## Background

Most Indigenous populations in colonised countries experience poor health outcomes relative to their non-Indigenous counterparts [1]. The poor health status of the Aboriginal and Torres Strait Islander population is well documented [2], with the life expectancy gap between Indigenous and non-Indigenous people one of contemporary Australia’s most enduring health divides [3]. Among the Australian Indigenous population, chronic diseases (CDs) are the greatest contributor to these health disparities [4]. For example, cardiovascular disease (CVD) is the single leading cause of death among Aboriginal and Torres Strait Islander people [3], and type 2 diabetes (T2DM) is at epidemic proportions [5]. Rates of chronic kidney disease (CKD) are also disproportionately higher among Aboriginal and Torres Strait Islander people compared with non-Indigenous Australians [6]. Collectively, these conditions account for up to 50 % of the life expectancy gap between Aboriginal and Torres Strait Islander and non-Indigenous Australians [7].

The role of primary health care (PHC), as intended by the Declaration of Alma Ata, includes promoting health, preventing disease and managing the poor health of local populations by maximising the use of local resources [8]. Access to appropriate, affordable, acceptable and comprehensive PHC is critical for improving the health of Aboriginal and Torres Strait Islanders [9] and Indigenous populations worldwide. In support of this, a recent study has shown that better access to PHC that is responsive to the needs of Aboriginal and Torres Strait Islander people has reduced the rates of avoidable hospitalisation among them [10].

The success of PHC services relies not only on the provision of sufficient resources. Whilst appropriate infrastructure, sufficient funding and knowledgeable health care professionals are crucial, these elements alone will not lead to the provision of appropriate care for all Indigenous people [11]. Low quality or racist treatment afforded to some Indigenous patients, for example, continues to discourage people from accessing services [12, 13]. Rather than solely emphasising the implementation of evidence-based treatment methods, research suggests that health care providers need to also understand health from the perspective of the patient, appreciate the importance of establishing long-term relationships with the community, provide an ‘Indigenous space’ where patients feel comfortable and cared for, and respect the strong ties that Indigenous people have to family and their land [14].

The objective of this systematic review was to gain a more comprehensive, evidence-based understanding of factors that support (enablers) and inhibit (barriers) the implementation of interventions aimed at improving CD care for Indigenous people within a PHC setting. Whilst a number of literature reviews have already identified a range of factors which impact upon the delivery of CD care in the general community [15–17], a preliminary search of several sources (Joanna Briggs Institute (JBI) Database of Systematic Reviews and Implementation Reports, The Cochrane Library, PubMed and PROSPERO) revealed that there was no systematic review either published or underway that considered this from the perspective of implementation of interventions which are designed to improve health care for Indigenous people living with a CD. A protocol outlining the objectives, inclusion criteria and methods of analysis for this review was published a priori to conducting the systematic review [18].

This systematic review aimed to identify and synthesise relevant international evidence on the factors that support or inhibit the implementation of interventions aimed at improving CD care for Indigenous people within the PHC setting.

## Methods

### Inclusion criteria

Participants were Indigenous people of any age with a CD, their family or community members, PHC providers (doctors, nurses, administrators, Indigenous Health Workers (IHW)), and policy and decision makers working in Indigenous health. Studies were included if they involved an intervention for the management of CVD, CKD, chronic respiratory disease (CRD), T2DM, mental health conditions/depression, and/or HIV/AIDS. Interventions were implemented in PHC settings in Australia, New Zealand (NZ), Canada or the United States (US). For the purpose of this review, a PHC setting was defined as those outside of the inpatient setting that patients could directly access, such as general practices, outpatient treatment and rural outreach services. Interventions of interest included any strategies designed to improve the effectiveness or accessibility of clinical care in the PHC setting for Indigenous people with one or more of the six CDs listed above. The phenomena of interest were participant perceptions of enablers and/or barriers, based on their attitudes, beliefs, expectations, understandings and knowledge, arising from their participation in CD preventative or management interventions. Qualitative studies, programme evaluations that support quantitative data collection with some qualitative inquiry, and descriptive studies, such as surveys, were considered for inclusion.

### Search strategy

Published and unpublished literature written in the English language was searched. Studies published from January 1998 to July 2013 were included in order to capture the introduction of evidence-based guidelines and systematic approaches to CD management. It is acknowledged, however, that the extent of guideline implementation and changes in the approach to CD care may have varied between countries during this time period. MEDLINE (PubMed platform), Web of Science, CINAHL, PsycINFO, EMBASE, ATSIHealth, Australian Indigenous HealthInfoNet via Informit Online, and PHCRIS were searched using key words and index terms that are provided in Additional file 1. In addition, the reference lists of all identified reports and articles were searched for additional studies.

The following search terms were applied to the databases (MEDLINE search shown in the following): (Indigenous[tiab] OR Aborigin*[tiab] OR Torres Strait Islander[tiab] OR Inuit[tiab] OR Maori[tiab] OR American Indian[tiab] OR Native American[tiab] OR First Nation[tiab] OR Oceanic Ancestry Group[Mesh] OR “American Native Continental Ancestry Group”[Mesh]) AND (Chronic disease[tiab] OR Chronic illness[tiab] OR Chronic respiratory disease[tiab] OR Obstructive lung disease[tiab] OR Chronic obstructive pulmonary disease[tiab] OR Bronchiectasis[tiab] OR Asthma[tiab] OR Cardiovascular disease[tiab] OR Heart disease[tiab] OR Atherosclerosis[tiab] OR Stroke[tiab] OR Arrhythmia[tiab] OR Heart attack[tiab] OR Myocardial infarction[tiab] OR Hypertension[tiab] OR Kidney disease[tiab] OR renal disease[tiab] OR Diabet*[tiab] OR depressi*[tiab] OR AIDS[tiab] OR acquired immune deficiency syndrome[tiab] OR HIV[tiab] OR Human immunodeficiency virus[tiab] OR Chronic disease[Mesh] OR “Respiratory Tract Diseases”[Mesh] OR Cardiovascular disease [Mesh] OR Kidney Diseases [Mesh] OR Diabetes Mellitus [Mesh] OR Depression [Mesh] OR Depressive Disorder [Mesh] OR HIV[Mesh] OR “HIV Infections”[Mesh]) AND (Primary health[tiab] OR primary care[tiab] Community[tiab] OR Outpatient[tiab] OR rural[tiab] OR Remote[tiab] OR Outreach[tiab] OR intervention[tiab] OR program*[tiab] OR ambulatory[tiab] OR general practice[tiab] OR “Health Care Quality, Access, and Evaluation”[Mesh] OR “Primary health care”[Mesh] OR “Health Services, Indigenous”[Mesh]).

### Study selection

Study selection was performed by four authors (KL, EA, CL, DR). The title and abstract of retrieved citations were reviewed against the inclusion criteria. Papers retrieved in full text were assessed against the review inclusion criteria by one reviewer (EA). When doubt arose, study eligibility was determined by discussion with the review team.

### Assessment of methodological quality

Papers that met the inclusion criteria were independently assessed for methodological quality by two reviewers using the appropriate (i.e. based on study design/type of study) standardised critical appraisal instruments from the Joanna Briggs Institute System for the Unified Management, Assessment and Review of Information (JBI SUMARI) (see Table 1 for appraisal criteria) [19]. Any disagreements were resolved by discussion between the two reviewers and, when necessary, were discussed with a third reviewer.

**Table 1:** Critical appraisal of included qualitative and quantitative studies

First author (year)	Q1	Q2	Q3	Q4	Q5	Q6	Q7	Q8	Q9	Q10
Critical appraisal of qualitative studies included in the review										
Wakerman (2005) [37]	U	U	U	U	U	N	N	N	Y	Y
Gardner (2010) [28]	U	U	U	U	U	N	N	N	N	U
Barney (2004) [40]	U	Y	Y	Y	Y	N	N	Y	N	Y
Lloyd (2008) [31]	U	U	U	U	U	N	N	Y	Y	Y
Lloyd (2009) [30]	U	Y	Y	Y	Y	N	N	Y	Y	Y
DiGiacomo (2010a) [26]	U	U	U	U	U	N	N	Y	Y	Y
Bailie (2004) [20]	U	U	U	U	U	N	N	N	Y	U
DiGiacomo (2010b) [27]	U	Y	Y	Y	Y	N	N	Y	Y	Y
d’Abbs (2008) [24]	U	Y	Y	Y	Y	Y	N	Y	N	Y
Thompson (2009) [35]	U	U	U	U	U	N	N	Y	Y	Y
Porter (2009) [39]	U	U	U	U	U	N	N	N	Y	Y
Ratima (1999) [38]	U	U	U	U	U	Y	N	Y	Y	Y
Si (2006) [34]	U	U	U	U	U	N	N	N	Y	U
Battersby (2008)	U	U	U	U	U	N	N	N	Y	Y
Barnett (2011) [21]	U	U	U	U	U	Y	Y	Y	Y	Y
Davidson (2008) [25]	U	U	U	U	U	Y	N	Y	U	Y
Carey (2013) [23]	U	Y	Y	Y	Y	Y	Y	Y	Y	Y
Schierhout (2010) [33]	U	U	U	U	U	N	N	N	Y	U
Kowanko (2012) [29]	U	Y	Y	Y	Y	N	N	Y	Y	Y
%	0.00	31.58	31.58	31.58	31.58	26.32	10.53	63.16	78.95	78.95
Critical appraisal of quantitative studies included in the review										
Si (2006) [34]	Y	Y	U	Y	N/A	Y	N	U	Y	
Longstreet (2005) [32]	Y	U	N	U	N	Y	U	U	Y	
Reilley (2010) [42]	Y	Y	Y	N	N/A	N/A	N/A	U	Y	
Reilley (2009) [41]	U	Y	N	N	N/A	Y	N/A	U	Y	
Tracey (2013) [36]	N	N	N/A	N/A	N/A	Y	N/A	N/A	N/A	
%	60.00	60.00	20.00	20.00	0.00	80.00	0.00	0.00	80.00	

Si (2006) [34] mixed method study has been appraised using both qualitative and quantitative instruments. Critical appraisal criteria for qualitative studies: (1) Is there congruency between the stated philosophical perspective between the research and the methodology? Is there congruity between the research methodology and the (2) research question or objectives? (3) methods used to collect data? (4) representation and analysis of data? (5) interpretation of results? (6) Is there a statement locating the researcher culturally or theoretically? (7) Is the influence of the research on the research and vice versa addressed? (8) Are participants and their voices, adequately represented? (9) Is the research ethical according to current criteria or, for recent studies, is there evidence of ethical approval by an appropriate body? (10) Do the conclusions drawn in the research report flow from the analysis or interpretation of the data? Critical appraisal criteria for quantitative studies: (1) Was the study based on a random or pseudo-random sample? (2) Were the criteria for inclusion in the sample clearly defined? (3) Were confounding factors identified and strategies to deal with them stated? (4) Were outcomes assessed using objective criteria? (5) If comparisons were being made, was there sufficient description of the groups? (6) Was follow-up carried out over a sufficient time period? (7) Were the outcomes of people who withdrew described and included in the analysis? (8) Were outcomes measured in a reliable way? (9) Was appropriate statistical analysis used?

*Y* yes, *N* no, *U* unclear, *N/A* not applicable

### Data collection

Data were extracted from primary studies using the standardised data extraction tool within JBI SUMARI which is provided in Additional file 2 [19]. For each qualitative and quantitative study, a description of the intervention, participants, setting, study methods and authors’ conclusions were extracted. Findings from qualitative studies were, where possible, extracted as themes, with one or more supporting illustrations from the text. A small number of qualitative studies did not present clear themes and, where this occurred, findings were extracted from the narrative in the form of a definitive statement made by authors following discussion by two reviewers (KL, OG).

All qualitative findings were assigned a level of credibility according to the following criteria: (1) unequivocal (U)—evidence beyond a reasonable doubt, including findings that were matter of fact, directly reported/observed and not open to challenge; (2) credible (C)—findings that were, albeit interpretations, plausible in light of the data and theoretical framework; (3) unsupported (Un)—where the study author’s finding was not congruent with nor supported by identifiable data. Unequivocal and credible findings only were included in the meta-synthesis. The credibility level of all findings is presented in Table 1.

### Data synthesis

Qualitative research findings were meta-aggregated and a set of statements were made to represent that aggregation. Qualitative research findings were pooled using JBI-QARI [19]. Findings were grouped into categories that were created on the basis of similarity of meaning. Categories were then meta-aggregated to produce a comprehensive set of synthesised findings that could be used to inform evidence-based practice. Quantitative data was presented in narrative summary.

## Results

**Fig. 1:**
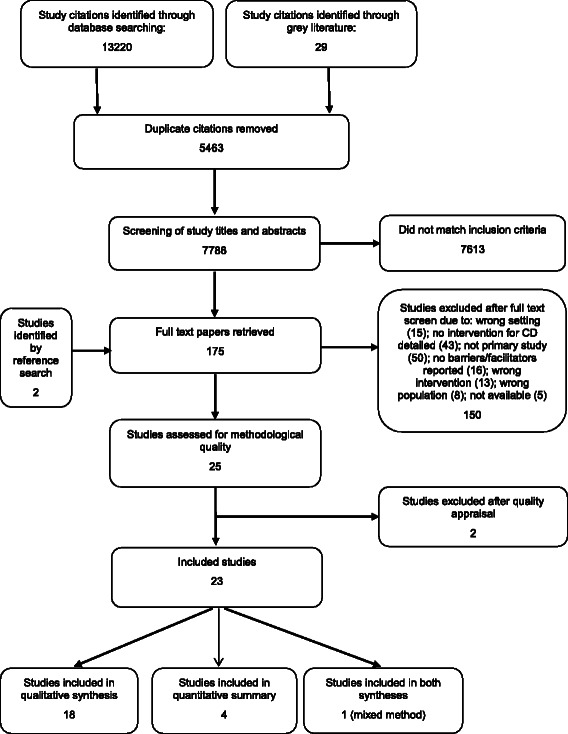
Flow diagram detailing results of literature search, study selection, assessment of methodological quality and synthesis

The search returned 7786 unique citations which were screened by title and abstract for congruency with the review inclusion criteria (Fig. 1). Following this initial screening, the full text of 175 papers was reviewed: 25 studies met the inclusion criteria and were assessed for methodological quality, 2 studies were excluded after appraisal, 23 studies were included for data synthesis, 18 studies used qualitative methods, 4 used quantitative methods, and 1 used a mixed method. Overall, the quality of the included qualitative and quantitative studies was moderate, mainly due to insufficient description of the methodological approach (Table 1).

Studies were conducted in Australia [20–37], NZ [38, 39] and the US [40–42]. No studies from Canada met the review inclusion criteria. Each of the six CDs of interest was represented: T2DM [20, 22, 32, 34, 39] or CVD [25–27, 35], mental health condition/depression [23], HIV [40–42], CKD [36] and CRD [38], and CD in general [21, 24, 28–31, 33, 37]. Participants included health professionals [20, 22, 24–27, 33–36, 41, 42], patients [38, 39] and policy and decision makers [37] or combinations of these [21, 23, 28–31, 40]. The characteristics of each study are shown in Additional file 3.

### Results from qualitative studies

From the 19 studies containing qualitative data, which included one mixed method study, 140 findings were extracted, encompassing both facilitators and barriers to the implementation of CD interventions in PHC. A list of findings extracted from included qualitative studies are shown in Additional file 4. These findings were grouped into 29 categories, which were then meta-aggregated into five synthesised findings (Table 2). The five synthesised findings provide an evidence-base that improves our understanding of factors that enable and inhibit the implementation of interventions aimed at improving CD care for Indigenous people in the PHC setting and therefore should be considered when implementing a CD intervention. The synthesised findings are:

**Table 2:** Meta-aggregated findings and categories

Five meta-aggregated synthesised findings and their categories
Finding: design attributes
• Partnering with Indigenous communities and individuals is critical to inform the design of an intervention in order to ensure the successful implementation and sustainability of the intervention
• Compatibility with systems and processes and adding value to existing services are qualities of a successful intervention
• Multiple funding sources could increase the budget to resource additional initiatives but this also increases reporting requirements. Funding accountability is closely linked to evaluation, which is imperative to build into the design of the intervention
• Local people employed as health workers are instruments for engaging patients
• There is a need for strong organisational and clinical leadership to effect change in chronic disease care through an intervention
• The context in which the intervention is implemented is critical in determining its success and sustainability. Context includes political will and the policy environment, the health service capacity for change and community acceptability
• Planning for sufficient workforce resourcing at the design stage is essential so that staff have the time they need to manage chronic diseases
• Willingness and capacity of existing staff to implement new ways of providing chronic disease care
Finding: chronic disease workforce
• Staff identify the need for cultural awareness training and that current training is not sufficient
• Chronic disease training and development for the primary health care workforce needs to be relevant and feasible
• Indigenous Health Workers are excluded from decision making and have limited support for carrying out their work
• Health care staff need support for their own well-being and to help them provide better care
• High turnover of staff in Indigenous health service provision precludes successful implementation of new initiatives
• Dedicated chronic disease positions with transparent roles and responsibilities will help ensure staff time is spent on chronic disease management
• There is a shortage of Aboriginal health workers in the primary health care workforce
Finding: clinical care pathways
• Lack of knowledge of available referral services prevents appropriate follow-up care
• Seamless referral pathways with a dedicated referral coordinator are important to avoid having patients fall through the gaps and miss out on care
• There are limitations in current clinical (electronic) support systems
Finding: patient/provider partnerships
• People with a chronic disease want to share responsibility for their care. They expect comprehensive information about their condition in lay language from service providers. Health care providers must allow patients to be a partner in their care.
• A role of the provider should be to support and empower patients to share responsibility for their health and health care.
• It is important that service providers can communicate in a culturally safe manner and relate to patients and the community
• It is important that service providers understand the competing demands and priorities of patients
Finding: access
• The presence of Indigenous health workers and/or the provision of health care in Indigenous spaces create a friendly and relaxed atmosphere for patients
• There are peer influences based on traditional or cultural understanding that can either enable or inhibit access to care
• Clients need to be able to consistently access services (i.e. ongoing service availability) and experience continuity of care
• Indigenous Health Workers provide cultural safety for patients
• It is important to consider the role of the family when providing chronic disease care to Indigenous patients and when considering the patient’s ability to manage their chronic disease
• The health care system is complex and requires an organised and coordinated approach to enable patients to navigate through it
• Providing a culturally safe service is an enabling factor for Indigenous patients accessing chronic disease management and must include treating patients and their families with respect and consideration

1. Design attributes: essential elements to consider during the design of a CD intervention in order to provide a solid foundation for successful implementation and sustainability. These include community engagement, the policy and funding environment, leadership, staff approach to change and sufficient resourcing.
2. CD workforce: workforce issues include difficulties recruiting and retaining staff, unsuitable workforce training and development, lack of dedicated CD positions with clear roles and responsibilities, excluding IHW from decision making, and the need to support staff well-being.
3. Patient/provider partnerships: the role of the provider extends beyond their professional and technical skills. Valued qualities of a CD health worker include being understanding, supportive and empowering, being able to communicate sensitively and allowing patients to be partners in their care.
4. Clinical care pathways: poorly performing electronic support systems and vague referral pathways are barriers to a service provider’s ability to deliver comprehensive CD care.
5. Access: access to CD care is facilitated by providing consistent services and coordinated care, embedding culturally safe work practices (for example, by employing local Indigenous people and providing care in Indigenous spaces and being influenced by patient perspectives related to beliefs and experiences regarding health care and family support.

### Results from quantitative studies

Only a small number of studies containing quantitative data were included in the review. Many quantitative studies investigated the effectiveness of CD interventions by measuring health outcomes rather than factors enabling or inhibiting their implementation and therefore did not meet the review inclusion criteria. Overall, the available quantitative data agreed with the qualitative data and were congruent with the synthesised findings described above (Table 3).

**Table 3:** Summary of quantitative findings

Quantitative findings
Design attributes
• Increased bureaucratic process and existing health system regulations were perceived barriers to intervention implementation; community education and financial support were enablers.
Chronic disease workforce
• Identified barriers included high staff turnover, demanding workloads, lack of staff training, lack of clear roles of CD staff and a lack of stable relationships between staff; facilitators included employing IHW and involving staff from all levels in intervention design and planning.
Clinical care pathways
• Improving patient referral, coordination and follow-up care.
Patient/provider partnerships
• Maintaining confidentiality.
Access
• Provision of culturally secure services and culturally appropriate education materials, an increase in the volume of services, and provision of transport and accommodation for patients from rural and remote regions were recognised as patient-related facilitators.

## Discussion

The purpose of the review was to identify enablers and barriers to implementing CD interventions in PHC settings that provide care to Indigenous peoples. Five synthesised findings comprising 29 categories of barriers and enablers emerged from the meta-synthesis. From these, a set of implications for practice was derived (Table 4). Whilst these five synthesised findings may also apply to other population groups in addition to Indigenous populations, a number of enablers and barriers within each finding specifically applied to interventions for Indigenous people as discussed below.

**Table 4:** Implications for practice

Practice implications
Design attributes
• Interventions should include a strategy for partnering with the community. This should include employment and training of local people to implement the intervention.
• Interventions should, where possible, be designed to be compatible with existing systems or processes, and/or provide training and support for staff. The intervention should add value to the service, in the form of gained knowledge or improvement in existing processes.
• Interventions must be adequately staffed to enable workers to complete their CD-specific tasks. Delegation of tasks and responsibilities of CD staff and the roles of all staff must be transparent to all workers.
• A strategy for impact evaluation must be proposed in the intervention design phase.
• A positive workplace culture should be fostered through strong leadership, with the presence of champions and change agents.
Chronic disease workforce
• Adequate and feasible training must be provided to staff to effectively implement the CD intervention. Cultural awareness training must be included.
• Indigenous Health Workers must be recruited, trained, employed, and included in all stages of the intervention.
• To mitigate high staff turnover in CD interventions, staff must be supported in their work. Reasonable workloads and adequate living conditions for remote staff should be considered.
Clinical care pathways
• A dedicated referral coordinator should be employed to bridge the gaps in referral processes.
Patient/provider partnerships
• Providers should receive guidance on how to communicate with their patients. Including patients in monitoring their progress and speaking with patients in lay language is important.
Access
• Indigenous health workers should be employed, and Indigenous people should be employed in other roles within PHC services. Where possible, services should be provided and delivered within culturally safe spaces.

### Design attributes

All five synthesised findings were relevant to the implementation and sustainability of a CD intervention; however, the design attributes are needed to be considered prior to implementation. Attributes to incorporate in the design phase of an intervention included obtaining sufficient funding, developing an evaluation framework, planning for adequate workforce resourcing, and engaging with communities.

Whilst sufficient funding enabled the implementation and sustainability of an intervention, Indigenous-specific services often needed to rely on a multitude of short-term government funding arrangements which threaten their sustainability and result in overwhelming reporting requirements [43]. Funding arrangements between Indigenous community-controlled health services and governments tend to be more complex than those between governments and general practice or tiers of government in Australia and elsewhere [44]. One of the key issues to be considered during the design phase is therefore adequate funding for both the implementation and sustainability of an intervention.

Findings from this review also identified the need to measure the impact of an intervention, whilst recognising this could be difficult in more challenging service environments. The lack of evaluation of interventions and, in general, PHC programmes in Indigenous health is a recognised limitation in the Australian health care setting [45]. Evaluation can improve service delivery and contribute to an evidence-base to inform policy decisions [46].

Community engagement was considered critical in the pre-implementation and planning stage to inform the design of an intervention and for sustainability. Involving communities in the design and implementation of interventions assists in ensuring that their particular health concerns are addressed in a culturally sensitive manner [47]. Sufficient workforce resourcing was also found to be crucial for successful intervention implementation, particularly where interventions relied on the capacity of an already overburdened workforce. In Australia, the largest proportion of the medical workforce is practicing in urban areas which has resulted in shortages in regional, rural and remote areas [48]. This indicates the need for innovative models of delivery to achieve adequate staffing in geographically isolated areas, as well as improved workforce incentives which may be beyond the scope of what an intervention can achieve.

### CD workforce

A factor particular to interventions intended for Indigenous people was the employment of local health workers who facilitated implementation. It was recognised in the literature that the roles of IHWs extended beyond those stated in position descriptions, a conclusion shared by them and their non-Indigenous colleagues. Indigenous Health Workers acted as cultural mentors to non-Indigenous staff and assisted in the provision of a culturally safe service. However, IHWs faced some unique barriers to participate in the implementation of CD interventions. More so than other professionals, IHWs found that the available training in CD management was not always relevant to the scope of their practice, and the skills they gained were not easily transferrable to other workplace settings. In addition, findings from this review suggest that these important members of the workforce were often excluded from decision making and lacked appropriate support for carrying out their work. As IHWs are particularly important for interventions intended for Indigenous communities, their participation in all levels of decision-making is crucial [49] and it is important to ensure they are not isolated, excluded or discriminated against in the workplace.

### Patient/provider partnerships

The significant emphasis on partnerships in the included studies may in part be due to the ongoing nature of living with and managing a CD. For Indigenous people, whether they live in Australia, New Zealand or the US, relationships with healthcare providers are particularly complex. This is primarily due to historical policies and practices that excluded Indigenous people from society [49]. These social injustices continue to have a profound effect on the lives of Indigenous people today [50]. The institutionalised discrimination and poor treatment of Indigenous patients by health care providers that still exists in many places today means that developing respectful and safe relationships with providers is particularly important for this patient group to ensure they are appropriately supported when seeking care [51]. A trusting relationship enables Indigenous people to access an intervention, and to achieve this may require more time, effort and understanding on behalf of the provider.

### Clinical care pathways

Two care pathways were clearly identified: one was within the local service and the other pathway was one that required access to referral services. Particular to Indigenous people was the lack of identification of Indigenous ethnicity at the point of care. Not having an Indigenous identifier in the state and national medical databases is thought to directly hamper efforts to meet the health needs of these people [52]. Findings pertaining to external clinical pathways also highlighted a lack of provider knowledge about external health services which could support improved health outcomes. It was clear that lacking awareness of other available services inhibited the ability of primary care providers to access the best possible care for their patients.

### Access

Access to an intervention was facilitated by employing IHWs, providing care in safe spaces and accepting the supportive role of family in a patient’s care. Employment of local Indigenous people facilitated access to services for Indigenous patients and their families. This finding is consistent with another study that found Indigenous patients were more likely to feel comfortable accessing care in a service where Indigenous clinical, reception, paramedical and/or administrative staff were employed [53]. Also healthcare provided in safe spaces that have physical and cultural meaning to Indigenous people facilitated access to care. Spaces can work to engage and empower people or to marginalise and suppress people [54]. In the mainstream health care system, spaces are often dominated by a Western perspective. However, with thought, time and energy, health care spaces can become less of a barrier and more of an enabler by ensuring that Indigenous people find them acceptable and welcoming. Finally, patients identified the supportive role of family in encouraging relatives to seek care and maintain the lifestyle changes often required for CD management. There are additional benefits that flow from involving family in CD care, including the possibility of younger generations having the opportunity to learn about CD risk factors, which could lead to them being motivated to adopt a healthier lifestyle [23]. However, it should be noted that the converse can also result. Not all community members have had positive experiences in accessing health care and this factor may, therefore, adversely influence their peers. Indigenous people’s encounters with health care services that have led to negative perceptions include feelings of not being taken seriously, having their personal circumstances disregarded and experiencing discriminating attitudes and behaviours from providers who have negative stereotypes about Indigenous peoples [55].

### Features of enablers and barriers

Following the meta-synthesis, three features relating to all 29 categories of enablers and barriers identified in this review emerged. Over and above the five synthesised findings, these three features further clarified important characteristics of the identified enablers and barriers.

### Drivers for change

The enablers and barriers identified by this review were not fixed but variable concepts, capable of moving along a continuum from an enabler to a barrier and vice versa, and in some cases fulfilling the role of both. Consider the complexity of multiple funding streams, for example. Whilst administratively, funding agreements with several organisations were identified as a barrier, receiving funding from multiple sources facilitated the purchase of additional resources. Equally, if staff, who were initially unwilling to participate in the intervention, could be persuaded that the intervention would be able to deliver real benefits to their community, this barrier could become an enabler. Therefore, rather than fixed concepts, it may be helpful to think of enablers and barriers as potential drivers for change, that require consideration during the planning/design, implementation and/or ongoing management of an intervention.

### Degrees of influence

Another feature identified in this systematic review was the degree to which PHC interventions could influence drivers for change. It appeared that drivers for change could be directly influenced through the design and implementation of an intervention in only certain circumstances. For example, the decision to employ an IHW is completely within the control of the intervention. Conversely, it is unlikely that any design feature could directly influence government funding models. This suggests that the work of an intervention would be better served by focusing on those drivers for change over which they had at least some influence.

### Inter-relatedness

The final feature identified in this review was that the drivers for change were likely to be inter-related. However, the extent to which they impacted on each other was not entirely clear, nor was the level of importance that could be attributed to any one of the drivers for change. For example, employing IHWs to help facilitate engagement with the community may contribute to creating a welcoming and comfortable atmosphere and assist with delivering a culturally safe service. However, employing IHWs could also exacerbate the issue of them being excluded from decision-making and having limited support for carrying out their work. Another systematic literature review found that in “difficult to service” communities (i.e. geographically rural and remote locations), workforce shortages became less of an issue when funding, governance, management and leadership, as well as linkages with community agencies and infrastructure were addressed [45]. The authors attributed this outcome to a systematic approach to addressing health system gaps, which they termed “environmental enablers” and “essential service requirements” of sustainable primary care services (p 120) [45]. This suggests that addressing some drivers for change may limit the negative impact of others, in particular those which may be potentially more difficult to influence.

### Limitations of the review

Even though a rigorous search strategy was employed, it is possible that relevant studies may have been missed. In addition, whilst the intention of the review was to evaluate literature specific to interventions conducted in Australia, NZ, Canada and the US, the vast majority of studies were undertaken in Australia, and no studies from Canada contributed to the review findings. Therefore, these results may not thoroughly reflect experiences in all of the predetermined countries of interest, particularly Canada. Studies that did not include an identifiable intervention but, instead, investigated experiences of patients with a CD, and provider or policy maker perspectives on CD services or care were also excluded from this review. Although these studies did not meet the inclusion criteria, their data may have contributed perspectives on what we have come to call “drivers for change”. Finally, much of the research in this review represented the perspective of the service provider, less so that of the patient and the community and, least of all, that of the policy/decision maker. Even though the findings from service providers often reflected organisational or interpersonal enablers and barriers, structural level factors such as governance, collaboration with funders, policy implementation and resourcing are unequivocally related. The inclusion of decision makers in the design, implementation and sustainability of CD interventions is necessary to help promote or redress structural enablers or barriers. Further research is required to make explicit the opinions and perspectives of policy makers to establish whether there are additional drivers for change and, hence, further actions required to enhance implementation.

## Conclusions

This is the first time that the question of enablers and barriers to the implementation of PHC interventions for Indigenous people with CD has been addressed by a systematic review. Five key findings—design attributes, CD workforce, patient/provider partnerships, clinical care pathways and access—were found to impact upon intervention implementation and/or sustainability within a PHC setting. These concepts are well-established in the literature and have continued to surface over the last 15 years. Consideration of the three key features of enablers and barriers that emerged from the findings of this review may assist with effectively addressing the drivers for change. As the findings suggest, genuine collaboration between the intervention team, service providers, Indigenous patients, the community and policy makers is essential to the design, implementation and sustainability of interventions that will result in improved health and well-being of the population they are intended for. With direction provided by a collective body of evidence, now is an opportune time to actively address these drivers for change (enablers and barriers), with the aim of moving forward existing and future CD interventions for Indigenous people.

## Additional files

Additional file 1:
**Search strategy. Detailed search terms used within each database.**
Additional file 2:
**QARI data extraction instrument. Instrument used to record the data extracted from each included qualitative article.**
Additional file 3: **Characteristics of included studies.** A succinct summary of each included study that describes the intervention, study participants and method. Additional file 4: **List of findings extracted from included qualitative studies.** Included qualitative study findings and their supporting illustrations.
