# Supplementary material for: Enablers and barriers to the implementation of primary health care interventions for Indigenous people with chronic diseases: a systematic review
Source: Implement Sci. 2015 May 22;10:71. doi: 10.1186/s13012-015-0261-x (PMC4465476; doi:10.1186/s13012-015-0261-x)
Supplement: Additional file 1: — Search strategy. Detailed search terms used within each database. [file 13012_2015_261_MOESM1_ESM.pdf]

Additional file I: Search strategy

MEDLINE (PubMed platform)

Search	Query
#1	Indigenous[tiab] OR Aborigin*[tiab] OR Torres Strait Islander[tiab] OR Inuit[tiab] OR Maori[tiab] OR American Indian[tiab] OR Native American[tiab] OR First Nation[tiab] OR Oceanic Ancestry Group[Mesh] OR "American Native Continental Ancestry Group"[Mesh]
#2	Chronic disease[tiab] OR Chronic illness[tiab] OR Chronic respiratory disease[tiab] OR Obstructive lung disease[tiab] OR Chronic obstructive pulmonary disease[tiab] OR Bronchiectasis[tiab] OR Asthma[tiab] OR Cardiovascular disease[tiab] OR Heart disease[tiab] OR Atherosclerosis[tiab] OR Stroke[tiab] OR Arrhythmia[tiab] OR Heart attack[tiab] OR Myocardial infarction[tiab] OR Hypertension[tiab] OR Kidney disease[tiab] OR renal disease[tiab] OR Diabet*[tiab] OR depressi*[tiab] OR AIDS[tiab] OR acquired immune deficiency syndrome[tiab] OR HIV[tiab] OR Human immunodeficiency virus[tiab] OR Chronic disease[Mesh] OR "Respiratory Tract Diseases"[Mesh] OR Cardiovascular disease [Mesh] OR Kidney Diseases [Mesh] OR Diabetes Mellitus [Mesh] OR Depression [Mesh] OR Depressive Disorder [Mesh] OR HIV[Mesh] OR "HIV Infections"[Mesh])
#3	Primary health[tiab] OR primary care[tiab] Community[tiab] OR Outpatient[tiab] OR rural[tiab] OR Remote[tiab] OR Outreach[tiab] OR intervention[tiab] OR program*[tiab] OR ambulatory[tiab] OR general practice[tiab] OR “Health Care Quality, Access, and Evaluation”[Mesh] OR “Primary health care”[Mesh] OR “Health Services, Indigenous”[Mesh]
#4	#1 AND #2 AND #3
	Limits: Publication date from 1998/01/01 to 2013/12/31; English language

Web of Science

Search	Query
#1	Indigenous OR Aborigin* OR "Torres Strait Islander" OR Inuit OR Maori OR "American Indian" OR "Native American" OR "First Nation"
#2	"Chronic disease" OR "Chronic illness" OR "Chronic respiratory disease" OR "Obstructive lung disease" OR "Chronic obstructive pulmonary disease" OR "Bronchiectasis" OR "Asthma" OR "Cardiovascular disease" OR "Heart disease" OR "Atherosclerosis" OR "Stroke" OR "Arrhythmia" OR "Heart attack" OR "Myocardial infarction" OR "Hypertension" OR "Kidney disease" OR "renal disease" OR "Diabetes" OR "Diabetic" OR "depressive" OR depression OR “AIDS” OR "acquired immune deficiency syndrome" OR “HIV” OR "Human immunodeficiency virus"
#3	"Primary health” OR "primary care" OR Community OR Outpatient OR Rural OR Remote OR Outreach OR intervention OR program* OR ambulatory OR "general practice"
#4	#1 AND #2 AND #3
	Limits: Timespan=1998-2013. Databases=SCI-EXPANDED, SSCI, A&HCI, CPCI-S, CPCI-SSH, CCR-EXPANDED, IC.

CINAHL

Search	Query
#1	Indigenous OR Aborigin* OR “Torres Strait Islander”OR Inuit OR Maori OR “American Indian” OR “Native American” OR “First Nation” OR (MH "Indigenous Peoples+")
#2	“Chronic disease” OR “Chronic illness” OR “Chronic respiratory disease” OR “Obstructive lung disease” OR “Chronic obstructive pulmonary disease” OR Bronchiectasis OR Asthma OR “Cardiovascular disease” OR “Heart disease” OR Atherosclerosis OR Stroke OR Arrhythmia OR “Heart attack” OR “Myocardial infarction” OR Hypertension OR “Kidney disease” OR “renal disease” OR Diabet* OR depressi* OR AIDS OR “acquired immune deficiency syndrome” OR HIV OR “Human immunodeficiency virus” OR (MH "Chronic

	Disease") OR (MH "Respiratory Tract Diseases+") OR (MH "Cardiovascular Diseases+") OR (MH "Kidney Diseases+") OR (MH "Diabetes Mellitus+") OR (MH "Depression+") OR (MH "Human Immunodeficiency Virus+") OR (MH "HIV Infections+")
#3	"Primary health" OR "primary care" OR Community OR Outpatient OR Rural OR Remote OR Outreach OR intervention OR program* OR ambulatory OR "general practice" OR (MH "Health Services, Indigenous") OR (MH "Health Services Administration+")
#4	#1 AND #2 AND #3
	Limits: 19980101-20130631; English Language

PsycINFO

Search	Query
#1	(Indigenous or Aborigin* OR "Torres Strait Islander" OR Inuit or Maori OR "American Indian" OR "Native American" OR "First Nation").mp. [mp=title, abstract, heading word, table of contents, key concepts, original title, tests & measures] OR exp Indigenous Populations/)
#2	"chronic disease" OR "chronic illness" OR "chronic respiratory disease" OR "chronic obstructive pulmonary disease" OR Bronchiectasis or Asthma OR "cardiovascular disease" OR "heart disease" OR atherosclerosis or stroke OR Arrhythmia OR "heart attack" OR "Myocardial infarction" OR hypertension OR "kidney disease" OR "renal disease" OR Diabet* OR depressi* OR AIDS OR "acquired immune deficiency syndrome" OR HIV OR "Human immunodeficiency virus" OR *Chronic Illness/ OR exp Respiratory Tract Disorders/ OR exp Cardiovascular Disorders/ OR exp Kidney Diseases/ OR exp Diabetes/ OR exp major depression/ OR exp "depression (emotion)"/ OR exp HIV/
#3	"Primary health" OR "primary care" OR Community OR Outpatient OR Rural OR Remote OR Outreach OR intervention OR program* OR ambulatory OR "general practice" OR exp Primary Health Care/ OR exp Health Care Utilization/ OR exp Health Care Delivery/ OR exp Health Care Policy/ OR exp evaluation/ OR *health care administration/ OR exp "quality of services"/ OR exp treatment barriers/
#4	#1 AND #2 AND #3
	Limits: English language and yr="1998 - 2013"

EMBASE

Search	Query
#1	indigenous OR aborigin* OR 'torres strait islander' OR inuit OR maori OR 'american indian' OR 'native american' OR 'first nation' OR 'indigenous people'/exp OR 'aborigine'/exp OR 'american indian'/exp OR 'maori'/exp OR 'eskimo'/exp
#2	'chronic disease' OR 'chronic illness' OR 'chronic respiratory disease' OR 'obstructive lung disease' OR 'chronic obstructive pulmonary disease' OR bronchiectasis OR asthma OR 'cardiovascular disease' OR 'heart disease' OR atherosclerosis OR stroke OR arrhythmia OR 'heart attack' OR 'myocardial infarction' OR hypertension OR 'kidney disease' OR 'renal disease' OR diabet* OR depressi* OR aids OR 'acquired immune deficiency syndrome' OR hiv OR 'human immunodeficiency virus' OR 'chronic disease'/exp OR 'chronic respiratory tract disease'/exp OR 'cardiovascular disease'/exp OR 'chronic kidney disease'/exp OR 'diabetes mellitus'/exp OR 'depression'/exp OR 'human immunodeficiency virus'/exp OR 'human immunodeficiency virus infection'/exp
#3	'primary health' OR 'primary care' OR community OR outpatient OR rural OR remote

	OR outreach OR intervention OR program* OR ambulatory OR 'general practice' OR 'health care quality'/exp OR 'health care system'/exp OR 'health care delivery'/exp OR 'disease management'/mj
#4	#1 AND #2 AND #3
	Limits: [english]/lim AND [1998-2013]/py

ATSIHealth via Informit Online

Search	Query
#1	Indigenous OR Aborigin* OR “Torres Strait Islander”
#2	“Chronic disease” OR “Chronic illness” OR “Chronic respiratory disease” OR “Obstructive lung disease” OR “Chronic obstructive pulmonary disease” OR Bronchiectasis OR Asthma OR “Cardiovascular disease” OR “Heart disease” OR Atherosclerosis OR Stroke OR Arrhythmia OR “Heart attack” OR “Myocardial infarction” OR Hypertension OR “Kidney disease” OR “renal disease” OR Diabet* OR depressi* OR AIDS OR “acquired immune deficiency syndrome” OR HIV OR “Human immunodeficiency virus”
#3	"Primary health” OR "primary care" OR Community OR Outpatient OR Rural OR Remote OR Outreach OR intervention OR program* OR ambulatory OR "general practice"
#4	#1 AND #2 AND #3

Australian Indigenous HealthInfoNet (conducted 25 July 2013)

Search terms
“chronic disease” and (“Primary health” or “community” or “outpatient” or “outreach”)
“chronic disease” and (“Primary health” or “community” or “outpatient” or “outreach” or “rural” or “remote”)
“chronic respiratory disease” and (“Primary health” or "community" or "outpatient" or "outreach" or "rural" or "remote")
“cardiovascular disease” and (“Primary health” or "community" or "outpatient" or "outreach" or "rural" or "remote")
“chronic kidney disease" and (“Primary health” or "community" or "outpatient" or "outreach" or "rural" or "remote")
“diabetes" and (“Primary health” or "community" or "outpatient" or "outreach" or "rural" or "remote")
“depression" and (“Primary health” or "community" or "outpatient" or "outreach" or "rural" or “remote”)
“HIV” and (“Primary health” or "community” or "outpatient" or "outreach" or "rural" or "remote")
“AIDS” and (“Primary health” or "community" or "outpatient" or "outreach" or "rural" or "remote")

PHCRIS

Conducted: 25 July 2013

Search engine: PHCRIS E-Bulletin

Categories: Reports (only)

Search terms
Indigenous and “chronic disease” and “primary health care”
Aboriginal and Torres Strait Islander and “chronic disease” and "primary health"

Indigenous and “chronic disease” and “outreach” | “outpatient” | "community"
Indigenous and “chronic disease” and “rural” | “remote” | "intervention"
Indigenous and “cardiovascular disease” and “primary health”
Indigenous and “kidney disease” and “primary health”
Indigenous and “respiratory disease” and “primary health”
Indigenous and “diabetes” and “primary health”
Indigenous and “depression” and “primary health”
Indigenous and “HIV” and “primary health”
Indigenous and “AIDs” and “primary health”
