# Supplementary material for: Enablers and barriers to the implementation of primary health care interventions for Indigenous people with chronic diseases: a systematic review
Source: Implement Sci. 2015 May 22;10:71. doi: 10.1186/s13012-015-0261-x (PMC4465476; doi:10.1186/s13012-015-0261-x)
Supplement: Additional file 2: — QARI data extraction instrument. Instrument used to record the data extracted from each included qualitative article. [file 13012_2015_261_MOESM2_ESM.pdf]

Additional file 2: QARI data extraction instrument

JBI QARI Data Extraction Form for Interpretive & Critical Research

Reviewer ________________________ Date ________________________

Author ________________________ Year ________________________

Journal ________________________ Record Number ________________________

Study Description

Methodology

Method

Phenomena of interest

Setting

Geographical

Cultural

Participants

Data analysis

Authors Conclusions

Comments

Complete Yes ☐ No ☐
