# Supplementary material for: Enablers and barriers to the implementation of primary health care interventions for Indigenous people with chronic diseases: a systematic review
Source: Implement Sci. 2015 May 22;10:71. doi: 10.1186/s13012-015-0261-x (PMC4465476; doi:10.1186/s13012-015-0261-x)
Supplement: Additional file 3: — Characteristics of included studies. A succinct summary of each included study that describes the intervention, study participants and method. [file 13012_2015_261_MOESM3_ESM.pdf]

Additional file 3: Characteristics of included studies

Characteristics of included qualitative studies

First author, date published (state, country)	Intervention and Health Condition	Phenomena of Interest	Methods	Participants	Setting
Bailie 2004 (NT, Australia)[20]	Implementing best practice clinical guidelines for Aboriginal people living with diabetes	Barriers to effective service delivery	In-depth interviews and observation	Service providers	Remote community health centres
Barnett 2011 (QLD, Australia)[21]	Stanford Chronic Disease Self-management Program implemented in Aboriginal communities for people with chronic disease	Acceptability of the intervention within Aboriginal communities and factors that could enhance engagement	Semi-structured interviews and focus groups	Total of 39 community members, service providers, carers, and peer leaders (who had participated in the course before becoming a leader)	Rural, regional and urban health centres
Barney 2004 (Alaska, USA)[40]	Case management for Alaska Natives living with HIV/AIDS	Elements of successful case management	Semi-structured interviews	Patients and their family, community members, service providers	Remote rural villages
Battersby 2008 (SA, Australia)[22]	A model of self-management support with Aboriginal people who have diabetes	Acceptability of the model for AHW and patients; barriers and enablers to completing the care plan assessments and supporting patient to achieve their goals	Focus group	Aboriginal Health Workers	Rural primary care service
Carey 2013 (Australia)[23]	A social and emotional well-being program for Aboriginal people needing social and emotional support	Issues of effectiveness and sustainability of the service	Semi-structured interviews	Service providers, service participants, carers, family members, referrers, intervention design staff	1 remote community primary health care service

Davidson 2008 (NSW, Australia)[25]	A collaborative model of cardiovascular education for AHW conducted within a partnership model	Relevance of course content, use of and barriers to the use of course information in the workplace and the course impact on practice	Semi-structured interviews	Aboriginal Health Workers from urban and rural areas	Metropolitan tertiary education campus
DiGiacomo 2010 (WA, Australia)[26]	Implementation of National Guidelines for Cardiac Rehabilitation and Secondary Prevention for Aboriginal and Torres Strait Islander peoples: a guide for health professionals	Perceptions of Aboriginal patients' access to cardiac rehabilitation and the role of institutional barriers in implementing the guidelines	Semi-structured interviews	Health professionals	Government health services and Aboriginal medical services across the state
DiGiacomo 2010 (WA, Australia)[27]	Implementation of National Guidelines for Cardiac Rehabilitation and Secondary Prevention for Aboriginal and Torres Strait Islander peoples: a guide for health professionals	Guideline implementation barriers; Perceptions of systems and processes following discharge from hospital, cardio rehabilitation referrals and identifying Aboriginal ethnicity and barriers to accessing cardio rehabilitation services.	Face-to-face semi-structured interviews	38 service providers	Government and community-controlled health services providing cardiac rehabilitation or secondary prevention to Aboriginal people in 10 rural and seven metropolitan areas
Gardner 2010 (NT, WA, NSW, QLD in Australia)[28]	Implementation of Audit and Best Practice for Chronic Disease, continuous quality improvement project	Factors influencing uptake and establishment of the CQI processes	Semi-structured in-depth interviews, observation and document review	48 service providers, policy officers, intervention team members	40 community-controlled and government primary health care services, in remote, regional and metropolitan areas
Kowanko 2012 (SA, Australia)[29]	Implementation of strategies to manage chronic conditions tailored to suit Aboriginal settings	Experiences and ideas about what works, what doesn't and why, in relation to approaches	Semi-structured interviews	18 patients and 12 staff	2 community-controlled and government primary health care services, 1 each in a metropolitan, regional and

	and clients	and management of chronic conditions. Benefits, barriers and enablers of chronic condition management strategies.			remote area
Lloyd 2008 (NT, Australia)[31]	Implementation of the Northern Territory Preventable Chronic Disease Strategy focused on diabetes, heart disease, hypertension, renal disease, chronic airway disease, in the Aboriginal population	Pathways, facilitators of and barriers to policy implementation in a complex health system	Semi-structured in-depth interviews (recorded)	Policy officers, service providers	Urban, rural and remotely located 'mainstream/government' policy or service delivery organisations
Lloyd 2009 (NT, Australia)[30]	Implementation of the Northern Territory Preventable Chronic Disease Strategy focused on diabetes, heart disease, hypertension, renal disease, chronic airway disease, in the Aboriginal population	Roles of professional values and the culture of the Australian health care system in facilitating and constraining implementation of the policy	In-depth interviews	Policy officers, service providers	Urban, rural and remotely located 'mainstream/government' policy or service delivery organisations
Porter 2009 (New Zealand)[39]	'Get Checked' program that provides free annual health reviews for all people, including Maori, with diabetes	Barriers to attending annual review	Phone interview or questionnaire	9 Maori/Pacific Islanders	8 Rotorua General Practice Group Primary Health Care Organisations
Ratima 1999 (New Zealand)[38]	Asthma self-management program for Maori people	Experiences of taking part in an asthma self-management program, specifically the acceptability of the program and aspects of the program that could be	Standardised, open-ended interviews by a Pumanawa Hauora researcher	47 Maori people living with asthma	Health clinics in traditional Maori community centres in rural Maori community

	improved.				
Schierhout 2010 (QLD, NT, WA, NSW in Australia)[33]	Audit and Best Practice in Chronic Disease extension project was the refinement and implementation of a systematic continuous quality improvement program	Perceptions of barriers and facilitators to implementation	Quarterly reports designed to elicit details on perceptions of barriers and facilitators; purposively structured dialogue with hub co-ordinators	Implementers, key informants	69 government, community controlled primary health centres and general practices across rural, remote, small towns and urban settings
Si 2006 (NT, Australia)[34]	Implementation of best practice clinical guidelines in the form of chronic disease specific and age-specific care plans maintained on electronic information systems among Aboriginal people with diabetes and other chronic diseases.	Barriers to Aboriginal health worker involvement in diabetes and chronic illness care	Semi-structured interviews	Health managers, service providers	Remote primary care services: one community controlled, 1 government
Thompson 2009 (WA, Australia)[35]	Implementation of national guidelines for cardiac rehabilitation and secondary prevention for Aboriginal and Torres Strait Islander peoples	Barriers and facilitators of guideline implementation	Face-to-face semi-structured interviews that allowed for in-depth discussion.	24 Service providers	Government/mainstream hospitals and community-based public cardiac rehabilitation services located in 7 rural and 10 metropolitan areas
Wakerman 2005 (NT, Australia)[37]	Sharing health care initiative that aims to improve health service management and quality of life for people with chronic diseases.	Factors promoting and inhibiting the sustainability of the Initiative	In-depth interviews and a review of project and evaluation documents	1 community-controlled health board member, 1 project evaluator, 3 government representatives	Aboriginal community-controlled primary health care service, remote location
d'Abbs 2008 (far north QLD, Australia)[24]	Pilot of an evaluation framework designed to evaluate the implementation of	Understandings of health and health services and perceptions of health-related matters	Group discussions, semi-structured interviews, participant observation, 30	Patients, community members, service providers	2 remote state government primary health care services

	Indigenous Chronic Disease strategy in Aboriginal populations focusing on type 2 diabetes		randomly selected clinic charts audited		

Characteristics of included quantitative studies

Study	Intervention and condition	Methods	Intervention participants	Setting	Qualitative inquiry
Longstreet 2005 (QLD, Australia)[32]	Program for Aboriginal and Torres Strait Islander people with diabetes. Holistic, systematic and multi-disciplinary health care approach.	Pre-post audit of medical records and brainstorming	108 patient records from diabetes register in pre-audit, 99 records post. 7.8% of patients non-Indigenous in pre-audit with significant increase to 28.7% in post-audit.	Aboriginal community-controlled primary health care service, regional location	Service provider perceptions of barriers to patient care and self-management
Reilley 2010 (USA)[42]	Implementation of CDC guidelines of routine HIV testing. Separate written consent should not be required (general medical consent should be considered sufficient)	Cross-sectional survey and additional comments	All peoples aged 13 – 64 years who presented at the service	US, Indian Health Service, Metropolitan	205 service providers experiences of barriers to implementation and their perceptions of patient acceptance, counselling and consent requirements
Reilley 2009 (Tucson, Arizona, USA)[41]	Implementation of CDC guidelines for routine HIV testing among and Native American peoples	Standardized questionnaire that included open-ended questions	All peoples aged 12 – 55 years who presented at the service	US, Indian Health Service, Metropolitan	41 service provider perspectives on patient and provider acceptance and lessons learned in offering expanded HIV testing
Si 2006 (NT, Australia)[34]	Implementation of best practice clinical guidelines in the form of chronic disease specific and age-specific care plans maintained on	Three-year audit of patient records and health worker profile, observations and semi-structured interviews	137 Aboriginal people with type 2 diabetes from 7 remote community health centres in the Northern Territory.	1 community- controlled and 1 government remote primary care services	17 service provider perspectives of barriers to Indigenous health worker involvement in chronic illness care.

	electronic information systems among Aboriginal people with diabetes and other chronic diseases.				
Tracey 2013 (WA, Australia)[36]	The Goldfields Kidney Disease Nursing Management Program (GKDNMP) designed to improve care processes and service delivery for people with or at risk of chronic kidney disease, implemented in Aboriginal communities	Descriptive narrative of program implementation based on anecdotal evidence	Rural and remote Aboriginal communities in Australia, including the Goldfields region of Western Australia (WA)	1 hospital and 1 community-controlled health centre in regional areas, outreach to several remote community health centres	Authors’ perception of program implementation (no methods, participant numbers and no objective measurement of barriers and facilitators reported)
