# Supplementary material for: Enablers and barriers to the implementation of primary health care interventions for Indigenous people with chronic diseases: a systematic review
Source: Implement Sci. 2015 May 22;10:71. doi: 10.1186/s13012-015-0261-x (PMC4465476; doi:10.1186/s13012-015-0261-x)
Supplement: Additional file 4: — List of findings extracted from included qualitative studies. Included qualitative study findings and their supporting illustrations. [file 13012_2015_261_MOESM4_ESM.pdf]

Additional file 4: List of findings extracted from included qualitative studies

Findings for: Bailie, R. S. Si, D. Robinson, G. W. Togni, S. J. d'Abbs, P. H. N. - Medical Journal of Australia (2004); A multifaceted healthservice intervention in remote Aboriginal communities: 3-year follow-up of the impact on diabetes care[20]

Finding 1	Discontinuities in staffing (Un)
Illustration	None
Finding 2	Lack of ongoing training and support for the care coordination process (Un)
Illustration	None
Finding 3	Lack of development of relevant work practices (Un)
Illustration	None
Finding 4	Lack of capacity to monitor service activity through timely analysis of service data (Un)
Illustration	None
Finding 5	Reluctance on the part of patients to accept offered services when presenting for acute, non-scheduled services (Un)
Illustration	None

Findings for: Barnett, L. Kendall, E. - Health Promot J Austr (2011); Culturally appropriate methods for enhancing the participation of Aboriginal Australians in health-promoting programs[21]

Finding 1	Local systems and structures (U)
Illustration	'When medical advice or services are required from health professionals, gender made this impossible. "…the first one [doctor] was male and there was some things that I couldn't talk to him because he was a male and personally, myself, I was going through very strong pain and a lot of woman stuff and I couldn't very well talk to him."' (Indigenous course participant/leader, female) (p 30)

	'I don't know what other problems you can have with males when they've got this sort of problem, like arthritis, but you need to think especially about men when it comes to anger, fear, frustration. Because men are the ones that it's really hard to reach out to.' (Indigenous, health worker, female) (p 30)
Finding 2	Cultural traditions and knowledge (U)
Illustration	'…a lot of clients think they're a guinea pig for doctors. They say "Nup, we're not going to let a white man poison us – we're going to go to bush tucker or the health store, we're not gunna [going to] take no more medicine here"… it's a fact of life, a part of the culture, to have a strong belief in bush medicine.' (Indigenous, community Elder) (p 30)
Finding 3	Indigenous communication methods: The Murri Grapevine (U)
Illustration	'Peer influence was reported to be a particularly successful method of persuading community members to participate in a program. It was a strong motivator of behaviour and self-initiated treatment, particularly in relation to chronic pain management. "I remember when your [other participant] mum told my mum that this [product] is good and my mum went straight out and bought it, so they all go out and buy it."' (Indigenous, Health Worker) (p 31)<br>'I find that you need to target that respected group in the community to do it [health intervention] first so they fully understand it, so they can filter it through their community, 'cause they're a pretty close-knit community.' (Non-indigenous, nurse) (p 31)
Finding 4	Local Murri leadership and community participation (U)
Illustration	'I'm a big believer in community participation for the simple reason that people will come when they [community] people [and] the professional delivering it side-by-side and that's a good partnership to have happening in any community.' (Indigenous, Health Worker) (p 31)<br>'…get more lay people in the community, really it's their program. If we [health workers] take a step back and allow them to do their own courses, then it's their program.' (Indigenous, hospital liaison officer) (p 31)

Findings for: Barney, D. D., Rosenthal, C. C., Speier, T. - AIDS Educ Prev (2004); Components of successful HIV/AIDS case management in Alaska Native villages[40]

Finding 1	The case manager: knowledge and professional skills - Procuring Resources (C)
Illustration	‘It is the capacity to find resources for which there is no predetermined course of action. The two most dominant needs reported by clients were “money” and “transportation.” Clients were “so poor financially,” and there were “hardly any jobs.”’ (p 210)<br>‘[Clients] have to travel to Anchorage… If you don’t have the money, you have to depend on support groups and family… [You need to] collect donations. That gets expensive… [In addition to airfare] you have to think of lodging and meals and transportation, then the hospital bill if you don’t have money or Medicaid or health insurance…’ (Service provider) (p 210)<br>‘The case manager helped to find housing. One client “didn’t have running water or the sewer system,” necessities for maintaining basic cleanliness. The case management agency “bought the washer and dryer” for another family.’ (p 210)<br>‘Acquiring medical support systems for seriously ill clients was even more difficult. One service worker observed: “If you have AIDS and you want to be treated at home, actually pulling that off can be phenomenal even-just having the trained people who are willing to come in, take care of you and having the equipment that you need in your home.”’ (Service worker) (p 210)<br>‘When we allowed this one young man to die in the village, I felt like we were inadequate. We didn’t have what we needed for him… I felt like he was really short-changed and I told him that…we don’t have the appropriate facility here.’ (Native tribal officer) (p 210)<br>‘No one described an easy formula to direct case managers to the resources they need. Clients needed case managers to “help find adequate money” and equipment.’ (p 210 - 211)
Finding 2	The case manager: knowledge and professional skills – Enabling (C)
Illustration	‘Navigating available services can be next to impossible for clients: “[There are the] corporation dollars, and the Indian Health Service dollars, and the this dollars, and the that dollars… if I were an infected individual, I would feel like I were in a maze and didn’t know how to swim out…”’ (p 211)<br>‘Without a case manager’s help, clients can “get lost in the papers” and “red tape.”’ (p 211)<br>‘It’s very hard for the people to fill out the forms. They’re complicated forms. Have you seen the Medicaid forms? They’re huge. Nine times out of ten, they forget something…[and] they have to start all over again.’ (Non-Native service provider) (p 211)<br>‘Villagers sometimes became so frustrated that they did not “even try anymore for Medicaid, Social

	Security . . . and any other income.'" (p 211)<br>'[The case manager needs] to work things through for people, especially people who aren't used to muddling through the white culture and all of the processes involved in it… [It is] sometimes daunting, just to go grocery shopping, or get to the dentist's office.' (p 211)<br>'One villager commented that the case manager worked on "the living will, all that kind of stuff. Stuff that I…never heard of it."' (Villager) (p 211)
Finding 3	The case manager: knowledge and professional skills - Enabling: Clients reported facing discrimination due to their ethnicity and their illness. They needed the case manager to advocate for their interests both inside and outside of the village (C)
Illustration	'A client's family member described how a HIV-infected friend "was getting the run around" at Native facilities. (Client's family member) (p 211)<br>'…there's a whole medical institution set up in Alaska, at least in Anchorage, that is discriminatory.' (Service provider) (p 211)<br>'It's happened-somebody's come over [to the hospital], been in the late stages of AIDS, needed treatment... [He] came to our hospital on a weekend. We did not admit him...the doctor wasn't his doctor. He sent him to ANS. He went to ANS, and he was up there for three days before he was treated.' (p 211)<br>'[HIV-infected clients] were treated differently immediately at the hospital and facility... They said they were treated like they were contaminated. [They were] made to feel real different and isolated and immediately told they had to go to another facility.' (Native service provider) (p 212)<br>'People were sort of scared of 'em [clients] because everybody has mixed feelings about how AIDS is passed on... we were passing a video around the village... that [said] AIDS in its final form, when you're dying and stuff, can be passed on through your spt and through your tears.' (Villager) (p 212)
Finding 4	The case manager: knowledge and professional skills - Brokering and Referral (C)
Illustration	'Several service providers believed that clients did not "know all the agencies" or "who to contact" with their needs.' (Service providers) (p 212)<br>'…the case manager should ensure that clients have "a directory" that "says these are the different people you can call and this is what they do."' (Service providers) (p 212)
Finding 5	The case manager: knowledge and professional skills - Education: As one of the few local sources of

	information about HIV/AIDS, the case manager educated clients and their families (C)
Illustration	‘Clients reported that the information the case manager shared was very helpful.’ (Clients) (p 212)<br>‘Clients also expected the case manager to know about new drugs and treatments.’ (Clients) (p 212)<br>‘The case manager was someone for an “individual and his family to talk to” who knew “all the options.”’ (p 212)
Finding 6	The case manager: knowledge and professional skills – Communication (C)
Illustration	'[Effective communication] is hard depending on how sick you are [and] how much you really understand... You can [learn from the case manager], but sometimes she’s fast and you don’t understand everything she’s saying.' (Villager) (p 213)<br>'Sometimes maybe they [providers] don’t counsel with their level. [Providers use] a lot of words, they don’t understand, they just get confused. Maybe have somebody that can translate when it happens that people don’t understand English.' (Villager) (p 213)<br>‘One provider commented that the father of a client was “handicapped with his English” and speaking in his Native language helped.’ (service provider) (p 213)
Finding 7	The case management agency: Facilitating Conditions – Location (C)
Illustration	'The case manager herself agreed that improving case management would almost require you to “live in the village for a while.”' (Case manager) (p 213)<br>'...villagers and providers indicated that someone inside the village might have problems with neutrality and confidentiality: “A Native from [the village] would have a hard time because they’re part of their system And part of their family... Someone in that village is going to have trouble... due to the fact of who they are and what family they were born into. So I don’t think it would [work] for it to be one of the people in the village.”' (Village person) (p 213 - 214)<br>'[Finding the best location is difficult.] If you have two communities, [and] they’re on opposite extremes of the region, are you located in the middle, or are you located close to one community, or are you still located where you’re close to services so that you can actually hold the person’s hand and take him to the different service providers’' (Case manager) (p 214)
Finding 8	The case management agency: Facilitating Conditions – Coordination (C)

Illustration	'Lack of agency-level coordination may have inhibited the case manager's work. A service provider from outside the agency witnessed a "fragmentation" within the agency. She noted that the agency needed to have "more cohesiveness...or nothing will work." As a representative of another agency, she felt that the case management agency also needed to communicate with other agencies more effectively: "I would like to see some kind of more positive projection from [the case management agency] so that the rest of us aren't sitting down here confused as to "What do they do? What exactly is there? Why is [the case manager] not coming back anymore?"' (Case, manager) (p 214)<br>'Another service provider commented that they needed to work with the case management agency about "more flexible" payment. The lack of coordination reportedly left the "patient in the middle" and could lead to "duplicating services."' (Service provider) (p 214)<br>'I had one particular individual who had seen a medical provider in a nearby community for some of the pneumonia's and skin problems that he was having. And when he became really ill, he was flown up to Anchorage. But because Anchorage had no medical records, they said that it was his first opportunistic infection. We [case management] have hooked up a local doctor, to the Alaska Native Medical Center's computer system, so if they see a client that's Native, there's an agreement between the local hospital and ANS.' (Case manager) (p 214)
Finding 9	The case management agency: Facilitating Conditions - Support for Service Providers (C)
Illustration	'The case manager felt "anger, empathy, sorrow" and "complete, utter frustration." A service provider endured "hopelessness and helplessness."' (p 214)<br>'I have found that in the 2 years that I've worked up here... I have become really kind of emotionally numb... [someone] said that when you reach the level I'm at, you're kind of selling your soul. And I've had to think about that, a lot. Because you don't have the energy to really put forth what you could put forth.' (Case manager) (p 214)<br>'An alcohol and substance abuse worker in the villages advocated finding "creative ways to take care of the people who take care of... people": "…special education, building up of [caregivers]…I would burn out, if I wasn't experiencing decent success. That's a real danger. 'Cause then, they go in hopeless, and negative, critical, angry, or whatever. And if they come in that state, they're toxic. And you can't be that way, and it's tough to be up, sometimes."'(Alcohol and substance abuse worker) (p 214)
Finding 10	The case management agency: Facilitating Conditions - Confidentiality and prevention case management

	(C)
Illustration	'Villagers believed that HIV/AIDS information has to remain confidential. However, they also saw it as a "major problem" and a barrier to services. Unfortunately, in these very small communities, villagers believed that "eventually you'll know who has HIV or who has AIDS" regardless of agency efforts.' (p 214)<br>'Two individuals recognized the need to provide one-on-one case management services to uninfected, high risk individuals. Prevention case management can help to reach those people who "aren't being reached because... [they are] isolated" from the rest of the village.' (p 215)
Finding 11	Case manager: values and person capabilities - Support for Clients and Families (U)
Illustration	'Certain people started telling me that just because I have this illness does not mean I'll be dying tomorrow. That took me about a year to learn and understand. And within that year I tried to commit suicide three separate times.' (Client) (p 205)<br>'Clients and their families reported feeling "scared," "ashamed," "alienated," and "very much alone."'(Clients and families) (p 205)<br>'[We experience] shame, guilt… [We ask] how did I ever let this disease enter my body? [There's] a lot of doubt. And if they're homeless, well it's definitely hopeless.' (HIV client) (p 205)<br>'Clients might feel "alone with their problem, living in a village as opposed to in Anchorage."' (State public health worker) (p 205)<br>'A non-Native service provider believed that "it would be so daunting, to come from a small village, and to have a disease that affects people this badly."' (non-Native service provider) (p 205).<br>'In general, service providers were amazed at "how little" clients "valued themselves as people." One Native tribal official described how clients would "wait for me to come to share it with their families." Sometimes clients "stopped getting any medical attention" because they were "so mad." Clients could also feel "really embarrassed" when dealing with new symptoms like "sores and a rash on their face."' (Service providers) (p 205 - 206)<br>'All the times that I've called up and said, "fuck you, fuck you, fuck you," she ain't never once hung up. That's an outlet I need once in a while. And she's willing to provide that… [We] walk away kind of raw once in a while, but it helps me to realize I can let off steam and not expect disaster.' (Client) (p 206)<br>'Another client expressed the same need: "I don't cope with it very well. I push it down. And then when I'm running to [the case manager], she kind of gets it." Support and friendship from the case manager

	improved clients' confidence and helped them to be hopeful. (Client) (p 206 - 207)<br>'After the case manager visited clients, family members and service providers remembered seeing clients "walk taller" and look "happy."' (Family members and service providers) (p 207)<br>'[She gives] great support for families… She explains things to you about what's going on with this person, how might they be feeling, and you feel comfortable enough to dump on her how you're feeling and the grieving and dealing with death. And if you have any kind of questions about HIV/AIDS, she'll help you, she'll explain it to you the best way she knows how.' (Client family member) (p 207)<br>'These people needed help desperately. They needed desperately help with the patient, but also with themselves as individuals and as a group. [They needed someone]… to help them fix a meal, to throw the wash in the laundry, to unload the washer… to kind of keep things going for them. (Tribal Official) (p 207)
Finding 12	Case manager: values and personal capabilities - Helping, sensitivity and relating to others (U)
Illustration	'[The case manager] is our age group, she's not older, she's not younger… I just feel more comfortable with her because she can be just as crazy as we are, and she's human, and she doesn't act super professional where she's on this high horse. She's down at our level… She doesn't make you out to look stupid if you don't know. She'll say, "Hey, it's okay." She's got a great sense of humor.' (Family member) (p 207)<br>"A service provider emphasized the importance of being "nonjudgmental," and a villager noted that the case manager's "sense of humor uplifted" clients and that she "didn't look down on them." (p 208)<br>'There really needs to be a guy, who can talk with the guys…. It's not that they wouldn't trust a woman in a position, but sometimes I think guys just are more frank with guys.' (non-Native service provider) (p 208)<br>'The case manager attributed her own success and clients' ability to relate to her to the fact that she "kept coming back" and "stuck it out."' (Case manager) (p 208)<br>'One service provider noted that it takes a long time to "get a real rapport with people in the village" and emphasized that "you cannot keep changing people."' (Service provider) (p 208)<br>'One villager praised the case manager for being "all fired up and just raring to go." He continued to explain that she had "given a hundred and ten percent" and that she was "a pretty brave person." Hard work earned the respect of clients and families.' (p 208)<br>'A non-Native provider explained that before you can provide help to a resistant community "you have to get the elders to support you."' (non-Native provider) (p 208)<br>'[In] certain villages you got to be aware of if they're Protestant, or if they're Catholic, or if they're Mormon,

	or if they're Bahai's, and what their beliefs are in [regards to] contraception. Do they believe in using the condom for self-protection, as far as sexuality goes?' (Client) (p 208)
Finding 13	Case manager: values and personal capabilities - Empowerment, Access, and Cooperation (C)
Illustration	'The part of the message I did not hear, at all, was... what's their [HIV-positive individuals'] role in their responsibility to themselves and to their community? One thing that I believe in is if you do something for somebody, or to someone, and you exclude the with-them part, that you ultimately make them weak and dependent.'(Alcohol and drug worker) (p 209)<br>'A non-Native service provider saw "a real desire" on the part of community health aides to provide support from within the village. (non-Native service provider) (p 209)<br>'The case manager herself believed that training "case managers that live in the village to be part of the networking system" could improve services.' (Case manager) (p 209)<br>'I need to start speaking about [HIV/AIDS] myself to other people. [The case manager] and my sister have been trying to get me to do some speaking… People that are infected need to start speaking up and saying we're not any different than anybody else. We just got this disease. We're not out to hurt anybody. (Client) (p 209)<br>'Several providers acknowledged that client care had improved because the case manager worked "as a team member" and because she could "team up well."' (Service providers) (p 210)

Findings for: Battersby, M. Ah Kit, J. Prideaux, C. Harvey, P. Collins, J. Mills, P. - Aus J Primary Health (2008); Implementing the Flinders Model of self-management support with Aboriginal people who have diabetes : findings from a pilot study[22]

Finding 1	The most difficult problem for AHWs was lack of time because of the competing demands of clinical crises (U)
Illustration	There were delays in getting clients to services and in filling in forms, which many Aboriginal people could not read or understand. (p 71)
Finding2	…psychosocial needs… in most cases, disease-specific goal setting was against a backdrop of social problems that were perceived to be more important (C)

Illustration	'AHWs were confronted with the problems of overcrowding, inadequate housing, finances, drug and alcohol misuse, domestic violence and unemployment. The successful negotiation of these central issues by project staff was critical in maintaining the patients' confidence in AHWs; however, their inability to provide solutions also caused some patients to lose confidence in the system.' (p 71)<br>'Often the issues were extremely confidential, such as childhood sex abuse, marital conflict or grief related to recent family loss. Only modest services were available to deal with these complex issues; generally there was a reluctance to use non-Aboriginal or mainstream services, which ultimately left the AHW to deal with the problems as best they could.' (p 71-72)

Findings for: Carey, TA - BMC Health Services Research (2013); A qualitative study of a social and emotional well-being service for a remote Indigenous Australian community: implications for access, effectiveness, and sustainability[23]

Finding 1	Funding: having different organizations involved provided an opportunity to get more funding than might have otherwise been the case (U)
Illustration	'On a positive note we were able to get funding for additional positions and we did.' (Stakeholder) (p 5)
Finding 2	Organizational factors: Different organizations were involved from the outset and this increased the complexity of such things as priorities, expectations, accountability, and reporting (U)
Illustration	'…there were four players in this program and there were four programs, all with different accountability and all with very uncertain timelines, so a dreadful mess.' (Stakeholder) (p 5)
Finding 3	Getting started: there was an awareness that developing the service within the community would take time. It was also acknowledged that luck and opportunism were involved as well as making use of existing relationships to recruit staff to the service (U)
Illustration	'We took care to ensure that everybody who needed to knew about it and, as I say, thought there was room for a new service. It was really just - obviously finding a suitable person to kick it off was always going to be difficult.' (Stakeholder) (p 5)<br>'The relationships that people build with community members are very slow and fragile in getting them

	going… We all know that service providers who attempt to provide from [a larger town] to remote communities do only touch the surface because they, unfortunately, can only drive in, have a meeting and drive out. Community members don't tend to work like that; they don't want to work like that so they will turn towards people who they know, who are residential in the community, who they're familiar with.' (Stakeholder) (p 5)
Finding 4	Funding: There were significant problems with funding provided by different organizations with different expectations (U)
Illustration	'often most of our white fella systems are really good at supporting us to get education or employment or whatever but they're all white fella things. It's about money or image and - whereas most of them want a sense of identity of who they are as Aboriginal people as well but there are very few opportunities that are funded to make that happen.' (Service provider) (p 5)
Finding 5	The future: Participants mentioned the need for capacity building within the community to increase the number of local people developing leadership roles (U)
Illustration	'Like I said I'd just like to see more jobs available for people that are on the ground in the community and like I said, communities have got different ways of dealing with their communities and we've got different cultures and the best way is to work with your own culture and that's where it'll bring that standard back…just to respect and show honour.' (Service provider) (p 6)<br>'The ideal would be to support other community workers to have that sort of role in the community and to really have an Indigenous response of support that they are really happy with and determine themselves.' (Service provider) (p 6)<br>'… to include a cost benefit sort of analysis so we can sell it to government as a significant new initiative which has benefits across a whole range of areas rather than the alternative fly-in, fly-out white experts into places.' (Service provider) (p 6)
Finding 6	Operational problems: There were problems with recruitment and also problems in the scope of the work required given the time available. Some participants had a sense that the demands of the position could leave the service providers feeling exhausted and overwhelmed (U)
Illustration	'I'd like to see more…feet on the ground because I feel that sometimes it's just manic and…a very short

	amount of time to… work in.' (Referrer) (p 6)
Finding 7	Operational problems: communication barriers sometimes had to be negotiated if the service provider and the person accessing the service were from different skin groups or if there were differences in their abilities with the use of English and Indigenous languages (U)
Illustration	'…some people won't go…it's because they can't because of cultural reasons, because of the skin group, that they're that side but the others aren't covered.' (Significant other) (p 6)
Finding 8	Personal struggles: Participants described a range of different problems that the service addressed (U)
Illustration	'Yeah mainly my kids put a lot of pressure on me. We used to live all squashed up in one house and I had my sister-in-law with us and her nephews and nieces and my two kids, all squashed up - and my partner - and that was all making it hard to cope.' (Service participant) (p 6)<br>'…he got drunk and fight with families, with me… He was being abusive.' (Service participant) (p 6)<br>'…we worked out that over a two year period 30 people had either completed or attempted suicide over a two year period, out of a population of 600.' (Stakeholder) (p 6)<br>'Well in this community there was a lot of alcohol issues and drugs and suicide of young people, with their relationships and stuff.' (Service provider) (p 6)
Finding 9	Program activities: There was a sense of teamwork among the providers of the SEWBS, colleagues in the clinic, and other local groups. Having someone to talk to who was trusted seemed important (U)
Illustration	'I think because a lot of us Aboriginal people are pretty shy and don't really like talking to other family members so it's good to talk with someone that you don't know.' (Service participant) (p 6)<br>'Yeah we definitely work as a team? If I'm having difficulties getting my ideas across to a family or family group or person I'll talk to him [one of the service providers] and…we bounce ideas off.' (Referrer) (p 6)<br>'Talking about it made me think, like it sort of straightens you out and - like talking and planning ahead too…' (Service participant) (p 6)
Finding 10	Program activities: The various programs that people could engage in were also beneficial (U)
Illustration	'…the activities of providing meaningful employment and meaningful activities for people is certainly very much a part of trying to improve the general mental health of people, especially the men, and that helping

	them to think about enterprises and things that they might do should be part of this program and that the community development aspect is fundamental to it.' (Stakeholder) (p 6)
Finding 11	Measuring outcomes: Participants communicated an awareness of the importance of accountability in terms of being able to assess the impact of the service. At the same time, however, there was an acknowledgement of the difficulties of measuring the kind of different work that was required in a remote Indigenous community (U)
Illustration	'There's lots of things going on and to actually show that what you've done has actually improved the lives of these people is always a bit trickier…' (Stakeholder) (p6)

Findings for: Davidson, P. M. Digiacomo, M. Abbott, P. Zecchin, R. Heal, P. E. Mieni, L. Sheerin, N. Smith, J. Mark, A. Bradbery, B. Davison, J. - Aust Health Rev (2008); A partnership model in the development and implementation of a collaborative, cardiovascular education program for Aboriginal Health Workers[25]

Finding1	Benefits: knowledge and skills obtained in the course and how the material was relevant in their current workplaces (U)
Illustration	'I was able to go back and tell the doctors about the new guidelines.' (Aboriginal health worker) (p 143)<br>'This has been perfect for my job, it's what I do everyday. Just getting more information and being sure of what I am saying has been great.' (Aboriginal health worker) (p 144)
Finding2	Not all participants felt that the course addressed their needs (U)
Illustration	'Learning to do a blood pressure is one thing... It would have been better if things had been more integrated ... like I would have liked to have known things like how does an AMS work... how do you integrate an Aboriginal Health Worker in the system... what is the professional role and scope of practice?'<br>'Focussing on the patient journey would have been a good idea ... like how do you get a referral.' (Aboriginal health worker) (p 144)
Finding3	Transition to adult learning a challenge (U)

Illustration	‘You know I really regret missing that session... I didn’t go that day because I didn’t finish my assignment ... I didn’t think it would be OK to say I didn’t have time or I needed help or something like that.’ (Aboriginal health worker) (p 144)<br>‘You know it was just full on from the start...I found that a bit tough... getting used to doing assignments and homework again... fitting it all in was you know a bit hard... I don’t know if I really expected all of that... even though I got all the stuff’ … it may have been better to get it all together... know what you we were really in for.’ (Aboriginal health worker) (p 144)

Findings for: DiGiacomo, M. L., Thompson, S. C., Smith, J. S., Taylor, K. P., Dimer, L. A., Ali, M. A., Wood, M. M., Leahy, T. G., Davidson, P. M. - Aust Health Rev (2010); 'I don't know why they don't come': barriers to participation in cardiac rehabilitation[27]

Finding 1	Lack of awareness of community settings (U)
Illustration	'It is such a different world up here compared with anywhere else and you couldn't possibly expect someone to understand the sorts of challenges that a person is going to face when they get back up there.' (Health professional, regional hospital) (p 455)
Finding 2	Lack of awareness of barriers to access (C)
Illustration	'I don't know why they don't come.' (Health Professional, regional hospital)(p 455)
Finding 3	Need for staff cultural awareness (U)
Illustration	'The feedback I get from patients coming discharged from the cardiac units is that their bodies were exposed a lot, which to them they have to cope with, the openness of being in an ICU, maybe with gowns flapping, parts that normally aren't viewed being seen, catheterisations and everything. And the feedback is they say that the staff need more cultural training... the staff need to understand when a patient says no, they need to sit down and yarn with that person.' (Health professional, Aboriginal medical service) (p 455)
Finding 4	Insufficient time allocation and depth of material in cultural training (U)
Illustration	'There is so much information people need to get through in orientation that cultural awareness is given a

	time spot, and how can you possibly talk about all of the issues in cultural awareness in 15 or 20 minutes? It is not really cultural awareness, it's just some basic do's and don'ts.' (Health professional, Aboriginal health service) (p 455)
Finding 5	Insufficient content of cultural training (U)
Illustration	'What they were covering, people just did not find it useful at all, the content of the actual session was not worthwhile... they don't really cover what we want to know.' (Health professional, regional hospital) (p 455)
Finding 6	Preference and need for Aboriginal Health staff (U)
Illustration	'One of the most important things in providing health services to Aboriginal people is to actually work with the people who have the cultural awareness, and that is the health workers, of course, Aboriginal people themselves... The people want services to be provided by their own people.' (Health professional, Aboriginal health Service) (p 455)
Finding 7	Aboriginal Health Worker as cultural mentor (C)
Illustration	'Donna [Aboriginal Health Worker] would knock and say, "would you like us to come in or do you want to come out on the grass or the veranda?" She sort of knew how to not sort of encroach on their space and things like that.' (Health professional, regional health service) (p 455)
Finding 8	Aboriginal Health Workers need support (U)
Illustration	'...my opinion is in relation to having Indigenous, Aboriginal, health liaison officers, health workers, in organisations like tertiary hospitals, secondary hospitals where they are not well supported both from the point of view of their differences in relation to health and culture and their level of expertise, and their support in relation to being able to do their work in a non-hostile, safe environment. There has been a lot of attrition with Aboriginal positions being placed in hospitals and I can see why in that they actually are expected to deal with everything in relation to Aboriginal health. They are not given or valued to the level of other health disciplines and they are not well supported. They can't represent their community in that sort of environment, so they leave.' (Metropolitan health service) (p 455)

Findings for: DiGiacomo, M., Davidson, P. M., Taylor, K. P., Smith, J. S., Dimer, L., Ali, M., Wood, M.

M., Leahy, T. G., Thompson, S. C. - Qual Prim Care (2010); Health information system linkage and coordination are critical for increasing access to secondary prevention in Aboriginal health: a qualitative study[26]

Finding 1	Deficiency in referral systems: Gap in referral process due to lack of awareness of service (U)
Illustration	‘I think this is our biggest problem, they are not getting referred. Like, every single person that is referred to us we contact and we see in some manner. But, it’s probably our fault as well there is not a good relationship between us and the cardiologists in Perth or the surrounding areas, because people might not think the service is available, or they won’t do it. Yeah, there’s a huge gap.’ (Population Health Unit, Remote area) (p 21)
Finding 2	Referral systems require dedicated coordinator: Importance of referral person (U)
Illustration	'Important to the referral process is having a designated person in place to push/refer patients coming through the ED. Particularly given that there has recently been high GP turnover, so you can't necessarily depend on primary care to ensure continuity of care if they are planning to retire or move.' (Health service, remote area) (p 21)
Finding 3	Deficiency in referral systems: Gap in referral process between metro and remote AMS (U)
Illustration	Respondent 1: '...there is a gap between when they have treatment in Perth and then coming back here that there is a risk they could sort of slip through the net... in an ideal situation, you would visit someone in hospital. You know, you would have some sort of continuity before they are discharged. We can't do that and we don't know exactly what day they are getting back. There are issues around the continuity of care even though we should get a discharge summary.' (p 21)<br>Respondent 2: ‘Quite often what happens is they go from Derby to Perth and then they come back to the hospital and the hospital gets a discharge summary, but we [the AMS] don’t... So sometimes - and that is not only for cardiac, but for other clients that has happened - we have nothing because it has gone to the hospital because they are not aware that there is an AMS here usually.’ (AMS, Remote area) (p 21)
Finding 4	Referral systems require dedicated coordinator: Referral flowchart or referral contact sheet (C)
Illustration	'There are some missing links in the referral chain. Having a contact sheet or flowchart of the referral

	people might help.' (Primary Health, Remote area) (p 21)
Finding 5	Referral systems require dedicated coordinator: Dedicated link person between hospital and AMS (U)
Illustration	‘Trying to get a connection with AMS is the most important thing because that is where a lot of Aboriginal people get health care from. I mean, you would have to dedicate so much time I think to get that connection going, so I would say it really needs someone just dedicated in that role to try and get a link between the hospital and the AMS, because there is just nothing… there is just no linkage at all. There is that real divide.’ (Tertiary Hospital, Metropolitan) (p 21)
Finding 6	Documenting Aboriginal status: Reliance on stored data to determine Aboriginal identity (U)
Illustration	'I pull up every patient on TOPAS and TOPAS identifies if they are Aboriginal. So if they are not identified on TOPAS, then they are not identified unless I can tell because of my own experience with the patient, you know, if I have met the patient and they are clearly Aboriginal or if I know the name and I think they might be Aboriginal. But usually I have seen most of the patients in the ward, but there are certain ones that I don’t see on the weekends and things like that.' (Tertiary Hospital, Metropolitan) (p 22)
Finding 7	Documenting Aboriginal Status: Lack of system to record status (U)
Illustration	'I am pretty sure there is a section in there that says, “Indigenous, tick the box” or whatever, but no one ever does it. It is not a sort of a thing that we use. Obviously, there are some that you can tell straight away and then there are others that you are sort of, “Well could be, could not be; I don’t know” you know?' (Population Health Unit, Remote area) (p 22)
Finding 8	Multiple incompatible IT Systems (impact on referral systems): Un-linked systems within service impact on reporting (U)
Illustration	'It s very hard because our doctors will only use one system and the staff use the other system, and they don’t link... So we have a very difficult time as far as getting the information off the two systems. We do have a difficult time reporting some of our statistics. So we are hoping that there will eventually just be one, because we actually have the systems, plus we have notes.' (Remote AMS) (p 22)
Finding 9	Multiple Incompatible IT Systems (impact on referral systems): Referral delays due to incompatible IT

	systems across different areas (U)
Illustration	'We have issues with when people get referred from the medical centre, we don't have the doctors, or what they write in their notes there, so we have to either get the patient to sign like a consent for sharing information - and that takes a couple of days to actually get it through to our computer, their latest results and things like that - or we have to contact PathWest. So there is a bit of running around considering that if they referred to us, it should automatically be available to us to find... And the same with AMS, you know, I might see someone at AMS and then I might see them in the hospital, but I haven't got my notes that I have written on their computer system, so I don’t really know what I did with them last time or what we talked about. So, yeah, that could be improved too... One of the other things with the people is that they move around so much. So they might live in Burringurrah for like five or six weeks and then they will come into town, so their medical notes are everywhere. They are all over the place and no one really knows where they are.' (Population Health Unit, Remote area) (p 22)
Finding 10	Unmet clinical information storage and retrieval needs (IT systems design): Input only non-specific health data (C)
Illustration	'I think it is an easy system to put my stats into, but it doesn't actually tell us anything useful at the end of the day... So you have got the circulatory system, and that would generally go down as probably heart disease, which is kind of broad.' (Primary Health, Remote area) (p 22)
Finding 11	Unmet clinical information storage and retrieval needs (IT systems design): Lack of specific health data output (U)
Illustration	'Basically, the only thing you get out of it is how many people you have seen, and you can divide it up into ages and female/male, but you can't actually take out people who didn't arrive, cancellations. You can distinguish between areas. So, like I might have seen 12 people in Exmouth and five people in Denham or whatever, but you can't pull a lot of information like what you are seeing them for. So you enter in all these stats about, 'Oh I saw them for weight loss or nourishing diet' or whatever, but you can't pull any of that out again, so it is a bit restrictive... You can pick two (diagnoses) on HCARe, so you can divide it up. So you might have diabetes and weight loss, but you can't choose more than two... you seem to put in all this information and you can't bring anything out of it. You put in whether it is a new referral or whether it is a review, and you put in all this stuff but you can't actually find out how many new people you saw or how

	many reviews. It is all just collated. So, definitely issues with that. We have been trying to get new ones for ages.' (Population Health Unit, Remote area) (p 23)
Finding 12	Unmet clinical information storage and retrieval needs (IT Systems Design): Lack of IT capacity to store health data in remote areas (U)
Illustration	'They are having a lot of trouble at the moment getting it to the remote area clinics. I know that the nurses out there - - I mean I am documenting notes that haven't had anything written in for three years, and that person could have had a CVA, an MI, everything within that time. There is absolutely no record for that locum nurse who is out there to even know what this person's health issues are.' (Health Service, Remote area) (p 23)

Findings for: Gardner, K. L., Dowden, M., Togni, S., Bailie, R. - Implement Sci (2010); Understanding uptake of continuous quality improvement in Indigenous primary health care: lessons from a multi-site case study of the Audit and Best Practice for Chronic Disease project[28]

Finding1	ABCD [intervention] attributes (C)
Illustration	'In the series of interviews conducted for this research, we found broad support for the ABCD approach to CQI and considerable enthusiasm for the benefits that were perceived as arising from its use. There was a widespread perception that the system offered some distinct advantages over pre-existing quality approaches, training and technical support were available to assist services with implementation, and services could adapt the use of the processes and steps in the CQI cycle to suit their own environment and needs.' (p 4)<br>'The main initial concerns related to the amount of work the ABCD generated.' (p 4)
Finding2	ABCD [intervention] attributes: technical support (C)
Illustration	'Training and technical support provided by ABCD project staff was seen by stakeholders as critical for getting the project up and running in services.' (p 5)<br>'As the number of participating services has grown, the project has experienced difficulty in meeting demand for support.' (p 5)

Finding3	ABCD [intervention] attributes: transfer of knowledge (C)
Illustration	'Some stakeholders saw potential for transferring the knowledge gained from implementing the ABCD to other tasks within the organization.' (p 5)
Finding4	Active dissemination process: role of expert opinion, champions and change agents (U)
Illustration	'ABCD gave health service managers tools and authority to adopt new ideas. Champions can be effective but you need to give people authority to act. ABCD reports, especially the impact on intermediate outcomes, were very compelling.' (p 5)<br>'The ABCD project team took an active approach to influencing the opinion of key stakeholders as a means of facilitating uptake of the project.' (p 5)<br>'Many stakeholders at different levels of the system had to be engaged, and ABCD efforts in this regard seem to have had an important, though differential impact on influencing provider opinion.' (p 5)<br>'The project manager and hub coordinators played a key role in the initial engagement of services and community health boards, particularly in two jurisdictions where their experience in working in Aboriginal health services and their links with communities, particularly remote ones, gave people confidence that ABCD was viable in those contexts.' (p 6)<br>'General practitioners (GPs) were seen as difficult to engage, and of all groups GPs were the least likely to attend the system assessment and feedback sessions.' (p 6)<br>'Where GPs were enthusiastic about ABCD, they were more likely to play a role in reviewing data and developing strategies for improving care.' (p 6)
Finding5	Organisational Antecedents: Absorptive capacity for new knowledge (C)
Illustration	'Where there were key staff who had an interest, some experience and expertise in using data for performance improvement purposes, uptake of the tools and processes proceeded with relative ease, and there was greater enthusiasm for what could be achieved.' (p 6)<br>'Well established administrative and information systems were also critical.' (p 6)
Finding6	Organisational Antecedents: Leadership and management (C)
Illustration	'In one site that had strong leadership and vision for ABCD, staff attended workshops, listened to presentations, and the Board was sent on a study tour to learn about quality improvement. ABCD was

	included as a standing item in regular senior management meetings. At a later time, the manager went to considerable lengths to employ GPs with a chronic disease focus and an interest in being involved in CQI.' (p 7)<br>'Successful leaders engaged staff in building a shared organizational vision as well as in making sense of what ABCD would mean in relation to their own role.' (p 7)<br>'Where leaders did not play a central role in engaging staff in building an organizational vision or provide a high-level formal mandate to proceed, it was largely left to individual project managers to work with clinics on putting ABCD into place. This left the process more to chance and depended on the power and inclination of middle managers to support it… despite enthusiasm in many places, the process hung on individual interest and goodwill.' (p 7)
Finding7	Organisational readiness: Tension for change (C)
Illustration	'The poverty in which Indigenous people live in these communities, the constant flow of staff in and out, the lack of apparent improvement year in and year out, the constant on-call, the long working hours, and the uncertainty surrounding the best way to intervene was described by people in this study.' (p 7)<br>'Ironically, in places where one might expect the tension for change to be greatest, the capacity for introducing it may be lowest: "I think ABCD is a great idea. If I could get time to do it, it might even make my staff stay."' (p 8)
Finding8	Organisational readiness: Compatibility with existing health systems and processes (C)
Illustration	'Health centre managers reported an excellent fit between the ABCD tools and pre-existing service delivery and administrative systems for chronic disease care delivery, particularly in relation to recall, care planning, and record keeping.' (p 8)<br>'…in remote areas where staff turnover is high, core positions are often vacant or filled by agency staff, the audit system is seen as unrealistic and a major barrier to ongoing implementation by many managers.' (p 8)
Finding9	Organisational readiness: Power balances--supporters versus opponents (C)
Illustration	'…when opposition did occur, it was usually manifest in refusal to participate in the process.' (p 8)<br>'Where opposition came from clinical staff, implementation of actions that could lead to improvements in

	care was more likely to be affected.' (p 8)<br>'Where there was support from a manager or senior clinical or more supporters than opponents, [the intervention] proceeded more rapidly.' (p 8)
Finding10	Organisational readiness: Project management (C)
Illustration	'In some sites, they were hampered by opposition or ambivalence from clinic staff, persistent staff turnover… this caused delays and interrupted progress through the cycle.' (p 8)
Finding11	Initial establishment into clinics: Approach to change (U)
Illustration	'In a number of organisations, vision was accompanied by a mandate to proceed and a clear framework for implementation that could support good project management.' (p 9)<br>'…leaders set up internal structures…to support implementation and where they worked well, they brought people from different places in the system together to discuss progress, examine results… and debate over what needed to happen next…It created a sense of shared purpose.' (p 9)<br>'These organisations displayed a sense that things could be done.' (p 9)
Finding12	Initial establishment into clinics: Dedication of resources (U)
Illustration	'The ABCD principle is good. The workload is too high. It isn't feasible.' (p 9)<br>'Bigger clinics tended to have more human and other resources that could be cobbled together at times when needed to assist with implementation. Among smaller clinics in remote locations that are hundreds of kilometres from towns, there are fewer opportunities for this.' (p 9)
Finding13	Initial establishment into clinics: Hands-on-approach (U)
Illustration	'It has improved recording… We are much better on papers. And it has raised awareness about what is best practice. It's a point of reference and there isn't anywhere else to pick that up.' (p 9)<br>'Most coordinators encouraged health centre staff to do at least some audits, and these had a dramatic impact on people's understanding of what best practice was and what quality improvement was aiming to achieve at the clinical level… Everyone spoke enthusiastically about the benefits of this educative process.' (p 9)

Finding14	Initial establishment into clinics: Decision making (C)
Illustration	‘In some sites, hub coordinators retained responsibility… In some [other] places, control remained entirely centralized, staff perceived that ABCD was a regional concern, and they did not engage in any meaningful discussions about the way they went about their work.’ (p 9)
Finding15	Outer system context (C)
Illustration	‘…the broader policy and program developments at the national and state levels provided a conducive backdrop for developing and taking up ABCD… the time was right for ABCD.’ (p 9)

Findings for: Kowanko, I., Helps, Y., Harvey, P., Battersby, M., McCurry, B., Carbine, R., Boyd, J., Abdulla, O. - (2012); Chronic Condition Management Strategies in Aboriginal Communities: Final Report 2011, Flinders University and the Aboriginal Health Council of South Australia, Adelaide[29]

Finding 1	Accessible, appropriate and affordable health services: Transport (U)
Illustration	'Transport is a big issue. You can organise as many appointments with as many different people as you like but if people can’t get to their appointments, it’s not going to help, so transport is a big issue… It puts a lot of strain on our transport service to try and provide transport for the clinical services that we run here.' (Staff) (p 41)
Finding 2	Accessible, appropriate and affordable health services: Limited or absent service (U)
Illustration	‘I think that’s the biggest thing in the Riverland--transport. A lot of people haven’t got transport. Do you think that the Peelies Bus sort of helps by going to the towns? It does, it does, for the people who haven’t got transport, and the people who aren’t confident enough to go to their doctor.’ (Client) (p41)<br>‘Sometimes follow ups are an issue, cos there are breaks in between. It’s very hard to get your clients back. One [client] got angry when the doctor didn’t come last time, so he won’t go this time. …Or else the doctor’s there, but by the time [the client] gets down there the doctor says he’s waited too long and he’s gone. And [the client] gets there and it’s all locked up.’ (Client) (p41)<br>‘It’s quicker to go to the Peelies Bus and get an appointment done through that way because, if it comes from mainstream you’ve got to wait about three or four weeks to see a doctor…the only other way we can

	see a doctor is Emergency.' (Client) (p41)<br>'I think if they're got a new patient they need to talk to the patient a bit longer, especially new diabetic patients.' (Client) (p41)
Finding 3	Accessible, appropriate and affordable health services: Affordable services (U)
Illustration	'We make a lot of phone calls - talk to people - are you willing to do this on Medicare rebate? Do you charge a gap fee? If there's a gap fee, we don't use them.' (Staff) (p 42)<br>…and if they've got healthcare cards, so it's sort of an incentive, it takes that barrier away of the cost of medication for people and then they're more likely to actually take their medication.' (Staff) (p 42)<br>'A discount voucher for prescriptions dispensed on the Peelies Bus makes treatment for chronic conditions more affordable, and consultations are bulk billed.' (Staff) (p 43)<br>..If they're all signed up [to the Closing the Gap program] they can get free medications.' (Staff) (p 43)
Finding 4	Accessible, appropriate and affordable health services: Culturally safe service: A culturally safe service was an enabling factor for chronic condition management, lack of culturally appropriate service was a barrier (U)
Illustration	'I know that a lot of people in my family they won't go to the doctor unless something is badly wrong and they can see that something's wrong.' (Client) (p 43)<br>'Biases existed in [mainstream] health services and health providers that made it an imperative for PLAHS that they had to produce their own workers and are specialists (eg diabetes, alcohol and other drugs, mental health) as opposed to using existing services, this need was reinforced by the Aboriginal community.' (Staff) (p43)<br>'Some community members will not access them for political reasons, for family reasons, and would feel alienated from access to the services on offer, because the people in control at the time were not following a particular line.' (Staff) (p 43)
Finding 5	Accessible, appropriate and affordable health services: Culturally safe service: Many clients were not concerned with the Aboriginal status of their health service providers, as long as they were treated with respect and consideration (U)
Illustration	'That just don't count for me because they're there to help us, they're there to serve us, you know. They're

	there to help us and give us a better understanding of our sickness. So that's good we've got people there. That's good. I don't mind.' (Client) (p 43)
Finding 6	Clinical information management systems: Having an accurate computerised system to manage clinical records and client recalls was a key enabler of structured chronic condition management, whereas lack of such a system was a barrier (U)
Illustration	'Communicare's alright. It changes with every year. ...that's where the patients have got all their records saved and all their data and that.' (Staff) (p 44)<br>'We have everything on Communicare, so that is like a patient recall system. We have paperless notes here so everything's scanned into that program, all of their IMVS results come through there, doctors' notes are all in there, so it just collects. So there's different access by different people so like as a nurse I can see different things to what the health workers can but everyone can access the care plans. If they have someone present to the clinic then it's quite easy for them to open up their care plan and see what they need done. We also have that recall system so if they were due a HbA1c then as soon as they open up that file they can do the HbA1c because it's flashed in red.' (Staff) (p 44)<br>'Our drivers have got to pick up patients and bring them in. They document on Communicare. If somebody was not there when they went to pick them up, that's documented.' (Staff) (p 44)<br>'There is lots of stuff you could do beforehand like the medical part of the Care Plan you could fit it in and it would be fantastic if we could get the Care Plans into Communicare. If it becomes one of the templates, certain bits of information should just be automatically self-populated so that decreases the amount of paperwork that the Health Workers and other staff will be involved in, and I think that will make it easier to be involved in the logistics of the Care Plan. You see everyone understands at an intellectual level why it's good. If the actual implementation becomes tedious, it's hard. ... We might modify those [care plan] documents to suit our needs and ... go to Communicare and say put this in as one of templates for us, so that we can lessen the amount of this constant transcribing that we need to do.' (Staff) (p 44)
Finding 7	Clinical information management systems: Clients appreciated reminders about checkups and referral appointments, generated through the clinical information systems. Some clients also liked keeping track of their clinical progress by discussing Communicare data with the health care staff (U)
Illustration	'Sometimes showing them their results over time is encouraging, they can gain understanding of their disease progression.' (Staff) (p 45)

	‘It would help a lot ... for me, to look at it like that [as a graph] is easier than looking at it like that [a list of numbers].’ (Staff) (p 45)
Finding 8	Co-ordination and team care arrangements: Workers’ knowledge about other programs and services beyond their own immediate responsibilities and their willingness to recommend them to clients (U)
Illustration	‘You know, some of that’s knowing about other programs as well. Even if I don’t know about it I’ll say, look okay, I’ll put you on to somebody, or we’re doing this there, you know, promoting it. Every time you go out you’re promoting the organisation.’ (Staff) (p 46)
Finding 9	Lack of qualified Aboriginal health workers: Aboriginal health services often struggle to fill positions for Aboriginal health workers, and that many Aboriginal health workers lack formal qualifications (U)
Illustration	‘We’re constantly struggling with staff numbers. People leave to do other things and then you can’t replace them. It’s not easy. ... I think the Health Council hasn’t run the Primary Health Care training course for a while and so that’s really made a hole in the cohort of people, and we are really noticing it now... We put an ad in a month ago and we did not get one person out of that that we could actually interview. Not one. It’s really disappointing.’ (Staff) (p 49)<br>‘I’ve been doing this for years, trying to focus straight onto one thing but you can’t when you get short-staffed and you’ve got to go and do other things.’ (Staff) (p 49)
Finding 10	Lack of qualified Aboriginal health workers: There are many Aboriginal health workers in the SA workforce who have not yet completed basic training, and have little knowledge about physiology, chronic disease processes and indicators (U)
Illustration	‘So I think the health worker training, as good as it may be, I think they should scrap it and focus on training everybody to be enrolled nurses…. they're not getting taught properly the clinical stuff. (Staff) (p 49)
Finding 11	Staff training in chronic condition management as a key factor influencing the success or otherwise of any chronic condition management strategy introduced in health services (U)
Illustration	‘It would be good to train all the health workers and so we can all know what it’s all about and what they actually do with the care plan.’ (Staff) (p 49)

	'I'd like to see more young health workers come through into this program to become diabetes educators or work in the area of chronic disease.' (Staff) (p 50)
Finding 12	Staff training in chronic condition management: staff participation in training did not necessarily translate to clinical confidence or ability to implement what they have learned into practice (U)
Illustration	'I think that a lot of our Health Workers go off and do some training and so they've got a bit of paper. It does not mean that they either feel competent or confident, so there is something that needs to happen between them getting their bits of paper to get into a place where they feel comfortable, and that's across the board.' (Staff) (p 50)<br>'Health workers are able to do the care planning, but I really see it lacking in that, you know, the doctors need to be reminded a lot of the time to look at medication and look at what their blood pressures levels have been, and follow up on that liver function test, you know, all that knowledge that don't come with being a health worker.' (Staff) (p 50)<br>'Yeah, and I love the care planning idea but there is a real, this is a real negative thing and it's only my personal opinion but some of the nurses here have a real ownership thing of that group of clients and I think that needs to change, like you need to actually be able to delegate some of those things out to other people not just "You do all of it because you know that it's going to get done," sort of thing.' (Staff) (p 50)
Finding 13	Engaging the community: Staff members worked hard to promote their health services (U)
Illustration	'The people in the community have a misunderstanding of what these people in here do ...So there's a big misconception out there as to what these people are doing, and if they're doing anything good for them....I'm trying to get the Aboriginal people to come to the health service and make them feel welcome.' (Staff) (p 50)
Finding 14	Engaging the community: Being Aboriginal and having local community connections was advantageous for staff in engaging Aboriginal clients (C)
Illustration	'As soon as I mention my last name, like connection back, you're in... it's like that yeah.' (Staff) (p 50)
Finding 15	Engaging the community: PLAHS employed and trained a core group of Aboriginal health workers as a deliberate and ultimately successful strategy to engage Aboriginal clients (U)

Illustration	‘A decision was made to employ a core team of four Aboriginal health workers and, once established, the group supported each other and shared the load. All four were fully trained and qualified, and this was a key ingredient for their sustainability. ...We deliberately chose two men and two women to be in the team of four, for obvious reasons and we found that those together were trusted. …The community response to this approach was to use the Aboriginal health workers in preference to non- Aboriginal nurses or the GP as first-line contact. The Aboriginal health workers gained the respect of their clients, and were publicly spoken about with pride by members of the community.’ (Staff) (p 51)<br>‘I’m not just a diabetes worker in the organisation. I am an employee of the community that works in the area of health so it’s how I promote it to the community.’ (Staff) (p 51)
Finding 16	Engaging the community: A range of ongoing community-focused health promotion activities organised through PLAHS helped engage the Port Lincoln Aboriginal community with the health service (U)
Illustration	‘An interesting activity was setting up a women’s walking group, which helped the women to get together (the power brokers of the community). After a while the kids started to come along too, and this became an opportunity to teach them about bush tucker and the right things to eat.’ (Staff) (p 50)<br>‘There’s the beginnings of a Men’s Group and there’s a whole bunch of people who have just gone on a men’s camp around chronic conditions and men’s engagement with the health system. It’s just starting now. It’s early days. ...it’s been a mission trying to get men to come back.’ (Staff) (p 50)<br>‘Yeah, we’ve got a Men’s Group up there. We have between 10 and 15 blokes come every fortnight. ...We don’t dictate. That’s the only thing we don’t do, we just let them come to enjoy themselves and don’t think about anything, just get them out of the house for a couple of hours.’ (Staff) (p 50)
Finding 17	Engaging individual clients: the importance of building and maintaining a trusting relationship with the clients for any chronic condition management strategy to be effective (U)
Illustration	‘You have a good yarn. Sort of get them out of their shell a little bit.’ (Staff) (p 51)<br>‘So it’s about sitting down with somebody and listening to them, because people will talk about something else then they’ll slowly get around to it, and the reason’s probably down the bottom of the pit, not at the top. The community, they know that you’ve got time for them and you can support them or you’ll get someone that can support them.’ (Staff) (p 51)
Finding 18	Engaging individual clients: The down side of strong relationships between clients and staff is that clients

	may approach workers, particularly Aboriginal health workers who are also community members, out of hours, leading to blurring of professional boundaries and potential burnout (U)
Illustration	'You don't want them to be too dependent on you, because it'll be all the time. Monday to Friday 9-5 is okay. The weekend is my time.' (Staff) (p 52)
Finding 19	Engaging clients as partners in their own chronic condition management: having dedicated and committed staff was vital for effective client engagement in their own chronic condition management (U)
Illustration	'They started to come and check up on me and got me to come in, keep checking up on me all the time and we had a bloke here ... He used to come around to the place there and sit down and have a drink of coffee and we'd go through everything, "how do we feel and are we taking our medicines?" When he came around it was good.' (Client) (p 52)<br>'We need people who are dedicated. It's not easy work. It takes a lot of energy as well. It takes a particular type of person.' (Staff) (p 52)<br>'We aim for people to go on a care plan. Depends on how much people are willing to access the service, there are still barriers there, with people accepting their condition. Going slowly and building a rapport works best, so that they enjoy the process ...If you get the rapport and the trust, it's our responsibility to provide the care.' (Staff) (p 52)<br>'So, taking it step by step I suppose is the way to go and once you get their confidence I think you can look at addressing some of those issues. As we've seen today with some of our clients, they're taking that next step and they're willing to participate in further sort of management of their chronic illness.' (Staff) (p52)
Finding 20	Engaging clients as partners in their own chronic condition management: role [of staff] is to support and advise, not to tell clients what to do (U)
Illustration	'It's for us to just sort of say to them, if you have a chronic illness, I mean, you have to look out, you want to address it. That's giving them the power. I think it's giving them ownership as well. If you start saying you've got to do this, you do that, then they just back away.' (Staff) (p52)<br>'Yeah it's really the relationship and not standing up there and lecturing ... just on the same level, simple words... just be genuine that you do care for their health; just let people know that you care for their health, without jumping on their lap and saying, "I'm going to look after you", or something like that.' (Staff) (p52)

Finding 21	Encouraging and supporting clients (C)
Illustration	‘We try to promote that we are here to support, not to tell people what to do.’ (Staff) (p 53)<br>‘We don’t dictate.’ (Staff) (p 53)
Finding 22	Genuinely caring about the client (U)
Illustration	‘They need to know I’m being genuine, not just “it’s a part of my job”. Forget that, be a human being and let people know that you are there to help, just not another figure, another stat number or something.’ (Staff) (p 53)<br>‘Yeah, because they ring up, they check on you and, you know, it - to have someone that cares, you know, it makes a big difference. You’re not alone, you feel. And you’re not alone with diabetes anyway, because everyone has it. But when you first get diagnosed with it you feel like you’re isolated.’ (Client) (p 53)
Finding 23	Being available to discuss problems: Knowing that the workers were available to discuss issues was very helpful to clients (U)
Illustration	‘She’s always available and that’s a really good thing. If I’m coming in she’s there and she listens. ... And then she goes off and makes all the appointments for me to do things like go to see the podiatrist or the dietician or whatever from what we talk about because stuff happens. What’s really good is that she’s really helpful. It makes a difference.’ (Client) (p 53)<br>‘They know they’ve got support if they’ve got a bit of a problem, if they don’t understand something, that they’re supported.’ (Staff) (p 53)<br>‘People just don’t support one another enough, I think, and a lot of the support they get for themselves actually comes from staff in the health services because we make that time to sit down with them and have a cup of coffee and talk about some of the issues, where families these days, a lot of people just don’t do that anymore.’ (Staff) (p 53)
Finding 24	Keeping clients motivated: The support and encouragement provided by health service providers, particularly the Aboriginal health workers and nursing staff, motivated clients to be active partners in their own chronic condition management (U)
Illustration	‘Sometimes I didn’t even want to lift my feet to even come to the meetings, but when you’ve got a nice lady like [name], and she’s always smiling and saying, “Come on, we can do this”. You know, part of their

	positive attitude, like I took on theirs and I thought, "I can do this. Bloody oath I can." (Client) (p 53)<br>'At some stage they might come to a point where they hit a wall and they'll fall off the truck for a while ... and when they do fall off that thing we try to give as much support as we can, and when stuff like that comes at them, we're pretty supportive of that side, so yeah.' (Staff) (p 54)<br>'Yeah. Well, to make sure things are realistic, that you don't set yourself up to fail I suppose, because I think if you can get wins on the board, sort of, for a client, then that helps keep them motivated.' (Staff) (p 54)
Finding 25	Client knowledge of chronic conditions and how to manage them: Clients were eager for more information about their health and wanted a better understanding of chronic conditions and how to manage them (U)
Illustration	'So I think that coming to [the health service] has been a really good thing because it's the knowledge that we're talking about all the time. And that makes a difference because then I know what sort of things I can do to help.' (Client) (p 54)<br>'Just to get them to try to actually understand the illnesses is important to them. That's all they really need I reckon. Just education, just an understanding of what their illness is and then, once they know that, they've got a better understanding, they'll know what's good, what's not good, what's right, what's wrong.' (Staff) (p 54)
Finding 26	Client knowledge of chronic conditions and how to manage them: Clients said that health professionals did not always explain things in plain language or take enough time, leaving clients confused and ill-informed, and inhibited about asking for clarification (U)
Illustration	'Nobody wants to feel silly by saying, what does that mean. You walk out the door and you say well what does that mean, you know.' (Client) (p 54)
Finding 27	Commitment to lifestyle change: Being Strong: client commitment to long-term lifestyle change as a key part of managing their own chronic conditions (U)
Illustration	'I'm just going to meet it head on and do whatever it takes to make myself better.' (Client) (p 55)<br>'I thought well you have to make a change in your lifestyle so that's what I actually done.' (Client) (p 55)<br>'Nobody else can work for your health. You've got to do it yourself. You've got to work for it. So yes, anybody out there if you want to live a long life, healthy life, you work for it.' (Client) (p 55)

	'I'm old enough now to realise about my life. That's why I've wanted to make a change. I'm not naive and easily pulled to one side by other people's opinions.' (Client) (p 55)<br>'I believe in putting temptation out there to challenge me. I don't believe in… if I give up drinking I'm not never, ever, ever going to go in a pub, that's ridiculous, you're cutting meeting people and things like that, so yeah, it's my choices.' (Client) (p 55)<br>'Always be true to yourself, acknowledge your gains and your losses. You know, some days you lose, and some days you win, but don't let it get you down.' (Client) (p 55)<br>'Some days you, you know, when you've got to wake up and give yourself a needle every morning it tends to turn you off. I don't know, some days you're up and some days you're down. Yeah, so I just look at it like, you know, if I don't have that, have this needle, well then I won't be here to see my grannies or anything like that. So I think of long-term things where, I don't know, I want to make sure I'm alive at 50. I want to make sure I'm perfect at 50, I'm healthy and happy.' (Client) (p 55)
Finding 28	Commitment to lifestyle change: Managing medications: Clients with complex chronic conditions often had to take multiple medications, and commitment to taking them regularly for life was necessary (U)
Illustration	'[I don't use a dosette] at the moment, I probably will I think because I tend to forget. So what we've done is because I take [insulin] every single day at half past eight, it helps because I have to take all my other medications. I do it all at once and that has helped because by the time [name] gets ready to go to school it's half past eight, …we know that we have to have our medication and that's the way we've worked it, it's the routine.' (Client) (p 55)<br>'But before I was working I was walking and doing everything but I just had my mind set on my diabetes... I paid attention to it, I did for about a year and brought everything that was sky high right down but with work now everything is going out the window because all my eating times have changed, my insulin times have changed. Don't know if it's worth it.' (Client) (p 56)
Finding 29	Family and peer support: support from families and friends to make and maintain the lifestyle changes needed to manage a chronic condition (U)
Illustration	'You must have a supporting family… because you cannot just do it on your own. Especially if your life has been used to doing things one way, then you have to change. But it's what you have at home that makes you pull through.' (Client) (p 57)

Finding 30	Chaotic life: Staff and clients acknowledged many Aboriginal people do not prioritise their own health needs because there are so many other pressing issues to cope with (U)
Illustration	'If you have other problems in your household there's no way that a younger person is going to sit down and pay attention to their diabetes.' (Client) (p 58)<br>'You're trying to talk to someone about their problems but they've got other issues; it could be housing; it could be no money; electricity is going to be turned off or something like that; other issues come before the health.' (Staff) (p 58)
Finding 31	Role model: Clients who were managing their chronic conditions quite well spoke of being role models for their families (U)
Illustration	'I act as an elder's role of a… a good teacher. A lot of people can't do that, even older than me. A lot can't do that. I got knowledge, wisdom, and I've got spirit.' (client 14) (p 58)<br>'Yeah, well I saw my mum, dad and sister all pass away through diabetes, and I said to myself I had to change my life so I can – whoever's left in my family I can show them how to eat healthy.' (client 7) (p 58)
Finding 32	Financial pressure: the cost of lifestyle change was a barrier to managing chronic conditions (U)
Illustration	'How can you tell somebody to check their sugar levels if they haven't got the machine to do it? …How can you tell them to buy healthy foods or food for the whole family if they haven't got enough money?' (Staff) (p 59)<br>'But, you know the other thing that puts people off? Is to eat healthy your bill goes from $100 to maybe $180. It - my bill, that food shopping bill- has just doubled. And it's sad, because you're trying to look after yourself and eat healthy, and money is your first object before we do anything. And our bill's gone up like massive because we're buying the healthy vegies and stuff. You look for the cheaper version of vegies, but you can't get Black and Gold bananas, can you, you know?' (Client) (p 59)<br>'We find now we buy our food, our shopping and a lot of it is fresh vegetables and because we live on a farm we freeze everything so it's easier, so I'm not buying food every day I come to town or when I come in, I do it once a fortnight and it's a lot cheaper. I've found that we've saved a lot of money and it's good quality stuff like vegetables and of course we eat everything in the cupboard now because there was a lot of wastage before because "I don't want to eat that today, I'll go to town and buy something", we don't do that now, we eat what's in our cupboard.' (Client) (p 59)

Findings for: Lloyd J.E., Wise., M.J., Weeramanthri T. - Aus Health Rev (2008); Changing shape: workforce and the implementation of Aboriginal health policy[31]

Finding 1	Aboriginal Health Workers were excluded from the policy development and implementation processes (U)
Illustration	'[Aboriginal Health Workers are] asked to have input but rarely asked if they fully understand what the policy means, and in terms of implementing a lot of those policies you only see bits and pieces of it, you only have small involvement.' (Manager) (p 178)
Finding 2	Aboriginal Health Workers work under greater pressure from their families and the community generally than the non-Aboriginal health professionals, but they received less professional and practical support (U)
Illustration	'I don't get a lot of support...I have got a child ten months old and I've got a partner and we are staying in a small flat, she is starting to walk, and the flats are really small. We need a house to grow a family. You need a house not a flat; especially because it has just got two rooms. One room has got a kitchen, lounge and laundry and the other room has the toilet, shower and bedroom.' (Aboriginal Health Worker) (p 178) 'There aren't any champions for Aboriginal Health Workers in the bureaucracy, not that I can see. No one is speaking out and saying, "Look, we can't afford to lose these people". It is almost being seen to be written out of the script but I can't see how we can progress without them.' (Manager) (p 178)
Finding 3	...high staff turnover as a barrier to implementing PCDS - High staff turnover does not enable health professionals to build trusting relationships they need to have with policy officers and erodes communities, trust of health professionals and of health services in general (U)
Illustration	'And in the last year, we've had nine nurses go through one of the positions… So that kind of churn in the staffing really makes it very hard to maintain your chronic disease programs … We haven't been successful in finding someone who can come for more than a short time.' (Doctor) (p 178)
Finding 4	There were too few health professionals employed to meet the demands of providing acute and chronic care, and too few Aboriginal health professional, in particular (C)
Illustration	Almost all interviewees identified the lack of dedicated chronic disease positions (doctors, Aboriginal Health Workers and, to a lesser extent, allied health professionals) as a major barrier to the

	implementation of the PCDS. Many interviewees commented on the need to increase the number and proportion of Aboriginal people employed as health professionals. (p 179)
Finding 5	The commitment and support from health service managers was identified as facilitating implementation (U)
Illustration	'When you look at where the chronic disease program is working well, it is usually where the clinic manager [health service manager] is really supportive.' (Policy Officer) (p 179)
Finding 6	...dedicated positions were needed to develop and maintain effective chronic disease programs (U)
Illustration	'With having those staff dedicated to chronic disease, they are able to spend more time with the clients, they are able to go back and see them every day, whereas if you're relying on the acute clinical staff to do that, they get tied up with people with coughs and colds.' (Manager) (p 179)
Finding 7	...they [new staff] were often required to work in acute care and were not given sufficient, quarantined time for chronic disease management work (U)
Illustration	'Every health centre has an acute workload and there is an extra expectation which is almost universal, that all staff will participate in dealing with an acute workload and, as a result, even the staff members who are nominally employed by the Preventable Chronic Disease Program don't get anything like the amount of time that would be necessary to actually do that job.' (Doctor) (p 180)
Finding 8	...new roles can create tensions between dedicated chronic disease and acute care staff (U)
Illustration	'Sometimes if they [those working in the chronic disease positions] feel the clinic is really busy and if they are sitting and doing paper work or updating the cards [recall system] then they often feel from the other staff, that are working clinically, [that they] are thinking "How come you can sit and do that when we're busy?" (Manager) (p 180)
Finding 9	[there is a] gap between the current roles required by the health system and the skills and experience of the current workforce (U)
Illustration	'I think we are always going to have this contradiction between what we know, what I think generally is well accepted by public health people, as what is necessary, but not having the workforce or the resources to

	really implement it.' (Researcher) (p 180)<br>'The other thing with getting out and doing some education is ... how to actually do education programs. It is not something that I have done a huge amount of ... And I think we are being told what is needed, but not how to do it.' (Nurse) (p 180)

Findings for: Llyod, J., Wise, M., Weeramanthri T. and Nugus P. - J Health Serv Res Policy (2009); The influence of professional values on the implementation of Aboriginal health policy[30]

Finding 1	Different professions: different values: Nurses sought clarity about responsibility for health care and they saw the need for services to develop an integrated set of responsibilities to enable them to provide effective health care (C)
Illustration	'A clinic can only do so much, we can show where there are problems but you can't actually change things happening out there, the [food supplied through the] store or people's attitudes [to healthy behaviours]. They might like to just sit around gambling all day, or they might want to smoke.' (Nurse) (p 8)
Finding 2	Different professions: different values: Aboriginal health workers identified the difficulties that health services have in addressing underlying determinants of health as major barriers to improving Aboriginal health (U)
Illustration	'This household, kids keep coming in with sores, scabies, and you treat the kids and they go home and a month later they will come back with scabies'. When asked why this was a problem with this particular house, the health worker explained: 'Well it's over crowded. When asked what needed to happen if the children kept presenting with the same problem, the health worker explained: 'You'd have to treat the whole house, which usually happens. But you need to have a look at the house. Who out of the house is getting income? Are the kids going to school? Is the house kept tidy? How many people are living there? But even if you do find all that out there is nothing you can do if it is over-crowded because there are no other houses to split that house up.' (Aboriginal health worker)(p 8)
Finding 3	Different professions: different values: Miscommunication was identified by some participants as a barrier to effectively sharing responsibility for Aboriginal health. There is a perception that Aboriginal Australians and non-Aboriginal Australians have different communication styles (U)

Illustration	'Some people say yes, yes, yes and then do no, no, no. They say I am interested, you say well take those tablets then, and then [they are thrown] in the bin or garden as you go out. Not everybody votes with their mouth. Some people make themselves scarce when you're looking for them...that's just a mechanism that Aboriginal people use, whereas we might just say we don't want to go.' (Nurse) (p 9)
Finding 4	Different professions: different values: Some nurses feel a degree of frustration towards Aboriginal people's behaviour, their reactions, their health status (U)
Illustration	'I think we worry more about their health than they do.' (Nurse) (p 9)
Finding 5	Interactions between individual values and professional values: the circumstances of patients with chronic disease are diverse and... one of the responsibilities of doctors is tailoring care according to patient needs and social environments (U)
Illustration	'Some of the patients have houses, they have proper cupboards and safe places to keep medicines. They have an appropriate number of people living in the house, and they don't have chaos reigning. And at the other end of the spectrum you've got people living without any housing, living in the open with a chronic disease. So in the one circumstance you've got people who are quite capable of going to a pharmacy and collecting prescriptions and having a whole lot of boxes of tablets and taking them at the appropriate times at home. And they've got the literacy skills to do so. And at the other end, you've got people who are illiterate with impaired eyesight living in circumstances where, on any particular day, the police or the wardens may come and move them on down the river, or whatever, and they may lose all their possessions at any time. And you've got everything in between, so you need to be sensitive to where people are at.' (Doctor) (p 9)
Finding 6	Interactions between individual values and professional values: The doctor perceives that Aboriginal patients are non-compliant because they are not committed to their health (U)
Illustration	'The main barriers are compliance from the patients. Education is extraordinarily difficult. For example, I had a woman the other day, aged 25, she had a mitral valve replacement two months ago, she has systemic lupus erythematosus… She's got asthma, she's got pneumonia, she was given a powerful antibiotic, intravenously for 7 days. When I saw her last week… I suggested another antibiotic... [The next day] I rang the clinic and said 'how is she?' because she was almost suitable to be evacuated. 'Oh, she

	hasn't started them yet.' That surprised me, because I thought the staff in the clinic would have given them to her, at least initially. They had impressed on to her that she had to take them, but she couldn't be bothered.' (Doctor) (p 9)
Finding 7	Interactions between individual values and professional values: the responsibility of the health service (U)
Illustration	'We've got what we call: a recall safety net… we wait six months and print a list then of all patients, from our health service, with chronic disease who haven't been here in the last six months, and then we go and visit them. And talk to them, and find out why, and try and get them to re-engage in the health service... Also the other problem that we really had to get on top of-- doctors here started using the medications... as a way of getting patients to come back. So they'd [the doctors] only give someone four weeks of tablets and they'd say I want to see [the patient] them in four weeks, and so they'd only write the script for four weeks. And that meant that when patients didn't come back, they didn't get their tablets either. So we've got [doctors] them to accept they should be writing alike for most people. And if they [the patients] decide that we're too busy, they can't be bothered waiting to see the doctor, at least they are still taking their tablets. So the control had to be taken off the health professional.' (Doctor) (p 9)
Finding 8	The culture within the health care system: Focusing on patients' compliance with medication over-emphasizes patient responsibility and draws attention away from the circumstances that influence patients' health, service provider responsibility and government responsibility (U)
Illustration	'The limitation of compliance being seen solely as patients' responsibility is that it [this perception/attitude] enters into the dangerous territory of victim blaming, it doesn't account for other significant factors such as living conditions, it doesn't account for service provider responsibility such as the extent to which service providers involve patients and patient circumstances in treatment plans, and it doesn't consider the responsibility of government to provide accessible health services and whether or not this responsibility is met.' (Doctor) (p 10)
Finding 9	The culture within the health care system: Changing the culture of health services is a difficult and time-consuming task (U)
Illustration	'There was a lot of resistance from a lot of the older remote nurses and health workers, to the idea that your job was to run these systems and go out and chase people for follow-up. And before that there was

	very much a culture of you treated what walked in the door. It is not until you do an audit and you show that someone with diabetes has attended the clinic 50 times during the year and they haven't had a blood pressure or a blood test done, that you can sort of win people over and say… your opportunistic care methods are not working. Because that diabetic will always come in at 9am on Monday morning when the clinic is jam-packed and you're always going to say can you come back on Wednesday and we'll do your blood test and they are never going to come, unless you go out and invite them in on Wednesday. So that culture has shifted.' (Doctor) (p 10)

Findings for: Porter, T. Le Lievre, C. Lawrenson, R. - J Prim Health Care (2009); Why don't patients with diagnosed diabetes attend a free 'Get Checked' annual review?[39]

Finding 1	Too much medication expected to be taken (Un)
Illustration	None
Finding 2	The diet is difficult (Un)
Illustration	None
Finding 3	Disempowered in decision-making (Un)
Illustration	None
Finding 4	Won't go to the doctor unless something is wrong with me (Un)
Illustration	None
Finding 5	Costs e.g. of medication (Un)
Illustration	None
Finding 6	No community-based clinic identified as own (Un)
Illustration	None

Finding 7	Moving between Auckland, Sydney, Rotorua (Un)
Illustration	None
Finding 8	Caring for children and family members (U)
Illustration	‘My babies come first.’ (p 223)<br>‘My son is terminally ill, I’m babysitting at my moko’s, I put myself last.’ (p 223)
Finding 9	I don’t know much about diabetes (U)
Illustration	‘It’s very poor [knowledge], I’m koretake, I know it’s not good to eat chocolate but I’m naughty. It’s popular [chocolate] with Maori.’ (p 224)
Finding 10	Difficulty with transport (Un)
Illustration	None
Finding 11	Diabetes services are not available after 5pm; job makes access difficult (Un)
Illustration	None

Findings for: Ratima, M. M. Fox, C. Fox, B. Karu, H. T. Gemmell, T. Slater, T. D'Souza, W. J. Pearce, N. E. - Aust N Z J Public Health (1999); Long-term benefits for Maori of an asthma self-management program in a Maori community which takes a partnership approach[38]

Finding 1	Relaxed atmosphere (C)
Illustration	‘When you talk to a doctor you always tense up, you can’t talk freely. Need to talk freely before you can start to get better. You could do that on this program. You need to be able to communicate or things get worse and worse.’ (p 603)
Finding 2	Maori-managed services were generally perceived as friendly (U)
Illustration	‘I can communicate with my people better than I can with anybody else. I feel more comfortable with my

	own.' (p 603)
Finding 3	Participants tended to find Maori program workers easier to talk to, so they felt more relaxed (U)
Illustration	'More relaxed talking to your own people. Answers were more satisfactory. They treat you as part of the family. You're not just a person walking through the door.' (p 603)
Finding 4	Participants commented positively on the use of Marae for program clinics (U)
Illustration	'When you go to a hospital it adds pressure, but coming to Marae, our own home there was no additional pressure. It was easy.' (p 603)
Finding 5	The iwi (tribe) provided an access point to established Maori community networks, community credibility, and marae as a base for the clinics (Un)
Illustration	None
Finding 6	Access to Maori community networks facilitated the recruitment of participants into the study (Un)
Illustration	None
Finding 7	Community credibility was essential in making the program acceptable to the community and maintaining community commitment to the program (Un)
Illustration	None

Findings for: Schierhout, G., Brands, J., Bailie, R. - The Lowitja Institute, Melbourne (2010); Audit and Best Practice for Chronic Disease Extension Project 2005-2009: Final Report[33]

Finding 1	Health centres with less institutional support from health authorities could be assisted through the steps in the CQI cycle, but required greater levels of resourcing from the ABCD Extension project staff to achieve this (Un)
Illustration	None

Finding 2	Stakeholder engagement was enhanced by the past successes of the project, including the development of ‘support scaffolding’ comprising the web-based support system, regional hubs, and pre-tested tools and processes. A body of peer-reviewed research evidence supporting the approach also strengthened engagement of some stakeholders (Un)
Illustration	None
Finding 3	Health centres located in regions with good high-level support for the project had 100% completion of the feedback and action planning steps in the ABCD Extension cycle. Health centres from regions with less high-level support evidenced patchy implementation, with fewer centres completing the systems assessment and action planning steps (Un)
Illustration	None
Finding 4	The establishment of linkages and compatibilities between ABCD Extension and reporting and funding frameworks within the health system encouraged ongoing participation in the project (Un)
Illustration	None
Finding 5	The sound evidencebase on which the project was built (including quality of care measures based on evidence-based guidelines) was one of the key factors contributing to successful uptake of the project overall (Un)
Illustration	None
Finding 6	Regional-level factors including support systems for action planning and for collaborative work between staff across health centres and systematic approaches were also associated with improvements in quality of care (Un)
Illustration	None
Finding 7	Factors related to staffing (stability, commitment to CQI and so on) were associated with improved quality of care through the ABCD Extension project (Un)
Illustration	None

Finding 8	Factors external to the project, such as introduction of new Medicare Benefits Schedule items, were likely to have promoted interest in the project and greater commitment to evidenced-based care (Un)
Illustration	None
Finding 9	The focus on the overall system was appreciated by many stakeholders as a constructive way to initiate dialogue about areas that need improvement without apportioning blame (Un)
Illustration	None
Finding 10	One barrier to wider appreciation of systems assessment may have been the absence of complementary uses for the detailed data outside of the health centre. This is in contrast to the clinical audit tools, which were appreciated for their ability to extract data for higher-level reporting or special funding programs (Un)
Illustration	None
Finding 11	Effective implementation of the systems assessment was heavily dependent on the quality of facilitation available, and this was found to vary widely between regions (Un)
Illustration	None
Finding 12	It was found to be harder to implement the systems assessment well, or at all, in health centres that had few staff and limited means of covering for time staff spent participating in the process (Un)
Illustration	None

Findings for: Si, D., Bailie, R. S., Togni, S. J., d'Abbs, P. H., Robinson, G. W. - Med J Aust (2006); Aboriginal health workers and diabetes care in remote community health centres: a mixed method analysis[34]

Finding 1	Insufficient and discontinuous training of AHWs on use of clinical guidelines and computerised information systems (Un)
Illustration	None

Finding 2	Lack of a clear division of roles among health care professionals in the area of chronic illness care (Un)
Illustration	None
Finding 3	Lack of stable relationships with non-Aboriginal nursing staff, which influenced retention and performance of AHWs (Un)
Illustration	None
Finding 4	High demand for acute care, which limited opportunities for AHWs to be involved in chronic illness care (Un)
Illustration	None

Findings for: Thompson, S. C., Digiacomo, M. L., Smith, J. S., Taylor, K. P., Dimer, L., Ali, M., Wood, M. M., Leahy, T. G., Davidson, P. M. - Aust New Zealand Health Policy (2009); Are the processes recommended by the NHMRC for improving Cardiac Rehabilitation (CR) for Aboriginal and Torres Strait Islander people being implemented?: an assessment of CR Services across Western Australia[35]

Finding 1	Workforce: Aboriginal health workers are important in mentoring other health professionals by providing cultural insights into care of Indigenous people (U)
Illustration	'One of the most important things in providing health services to Aboriginal people is to actually work with the people who have the cultural awareness, and that is the health workers, of course, Aboriginal people themselves...the people want services to be provided by their own people.' (Health professional) (p 4)
Finding 2	Workforce: Existing AHWs faced limited infrastructure and support (C)
Illustration	'They can't represent their community in that sort of environment, so they leave.' (Service provider) (p 4)
Finding 3	Workforce: High turnover of non-Indigenous staff impacted on initiative sustainability (C)
Illustration	'I'm just relieving for J...' '...been here for three months' '...leaving in two weeks.' (Service provider) (p 4)

Finding 4	Cultural competence: Failure to appreciate reasons for poor participation (U)
Illustration	'I don't know why they don't come.' (Service provider) (p 4)
Finding 5	Cultural competence: Features of programs and services are not congruent with Indigenous clients' lifestyles, culture, commitments, and preferences (C)
Illustration	'Maybe we could run an ATSI-specific class, because they have this huge shame factor when they are with other people and stuff that they don't like doing.' (Service provider) (p 4)
Finding 6	Linkages: Few systematic processes for identifying Indigenous people, inadequate communication and referral upon discharge (U)
Illustration	'We don't know exactly what day they (Aboriginal patients discharged from hospital) are getting back. There are issues around the continuity of care even though we should get a discharge summary.' (Service provider) (p 4)
Finding 7	Linkages: Lack of awareness of available services in different areas (C)
Illustration	'It's probably our fault as there is not a good relationship between us and the cardiologists in Perth or the surrounding areas, because people might not think the service is available.' (Service provider) (p 4)

Findings for: Wakerman, J., Chalmers, E. M., Humphreys, J. S., Clarence, C. L., Bell, A. I., Larson, A., Lyle, D., Pashen, D. R. - Med J Aust (2005); Sustainable chronic disease management in remote Australia[37]

Finding 1	Context (C)
Illustration	Timing was also a critical contextual factor. The triad of problem recognition, available solution and conducive political environment was realised. The problem of chronic diseases was recognised internationally and nationally, as reflected in the National Health Priorities and the efforts of national non-government organisations. The congruence between national policy and local readiness was critical to sustainability. (p S65)<br>Implementation flexibility is critical to sustainability in this very challenging service environment. In high-

	need, cross-cultural settings, programs are vulnerable to individual idiosyncrasy, competition for scarce resources, limited skills and diseconomies of scale. (p S65)
Finding 2	Community engagement (C)
Illustration	…the community valued the service and the community was valued by the service - an essential synergy for program sustainability. Intersectoral linkages within the community can also contribute to sustainability. For example, despite diminished funding in one sector, activities generated by this kind of community-based project in other sectors - social club, school or store - may continue. (p S65)<br>Community Support Workers "were respected people within the community"; The project team was very "community focused". (p S66)
Finding 3	Systems flexibility and adaptability: High staff turnover at local health service level (U)
Illustration	'Staff continuity is particularly important in an Indigenous context: "…if you know somebody, and that somebody has been there for quite a while it just works so much better." (p S67)
Finding 4	Systems flexibility and adaptability: Australian Public Service organisational culture valuing rapid movement through positions along generic public sector career path (U)
Illustration	'…they are about up the ladder, and they'll do that by hook or by crook' (p S66)<br>At a national level, high staff turnover is linked to a culture that values rapid career advancement and is subject to frequent organisational restructuring. Over 18 months within the Department of Health and Ageing, there was a new chief executive officer, four different first assistant secretaries, seven assistant secretaries and five different directors in this area. This was highlighted by the unusual longevity of a key bureaucrat who was involved with the program over an extended period. '...it comes down to a sort of personal thing and whether someone is actually committed to it ...from a policy sense as well, it is most unusual for somebody like myself to be around in the same area for four years, but I have.' (p S 67)
Finding 5	Systems flexibility and adaptability: Frequent organisational restructuring (U)
Illustration	'So who knows what is going to happen by next year? I will probably tell you we've had another three changes of personnel' (p S66)

Finding 6	Information systems (C)
Illustration	Timely and appropriate information flow is essential to ensuring program responsiveness and sustainability. …Access to information is empowering and builds trust and recognition of the value of the different organisations working within a program. Informal communication, such as the 'brown paper bag lunches' within the Department of Health and Ageing, can be very effective. Community involvement in producing videos and pamphlets facilitated good communication at a local level. (p S67)
Finding 7	The human nature of health care and policy (C)
Illustration	'…not very seamless and [not] like a machine. It doesn't work that way at all. It is very haphazard, and at times you've got to pick up the vibes and hope you've picked up the right vibes. In this particular case we did pick up the right vibes.' (p S67)

Findings for: d'Abbs, P., Schmidt, B., Dougherty, K., Senior, K.. - Aust. J. Rural Health (2008); Implementing a chronic disease strategy in two remote Indigenous Australian settings: A multi-method pilot evaluation[24]

Finding 1	Self-management of chronic conditions: individual responsibility (U)
Illustration	'People might need some education about how to look after their bodies, but health is just common sense. People have changed, they want someone else to look after their health. People are forgetting that the responsibility for looking after your body is yours - it's in you.' (Patient) (p 72)
Finding 2	Self-management of chronic conditions [does] not always mean what health professionals want it to mean (C)
Illustration	'After a night out drinking, it's important to get your blood pressure done, to get some more blood pressure tablets.' (Elderly male patient) (p 73)
